# Supplementary material for: Evidence for a transfer-to-trap mechanism of fluorophore concentration quenching in lipid bilayers
Source: Biophys J. 2024 Jul 22;123(18):3242–56. doi: 10.1016/j.bpj.2024.07.026 (PMC11427787; doi:10.1016/j.bpj.2024.07.026)
Supplement: Document S2. Article plus supporting material [file mmc2.pdf]

Biophysical Journal
Article

Biophysical Society

Evidence for a transfer-to-trap mechanism of fluorophore concentration quenching in lipid bilayers

Sophie A. Meredith,[1,2] Yuka Kusunoki,[3] Stephen D. Evans,[1] Kenichi Morigaki,[3] Simon D. Connell,[1,2] and Peter G. Adams[1,2,*]
[1]School of Physics and Astronomy, University of Leeds, Leeds, UK; [2]Astbury Centre for Structural Molecular Biology, University of Leeds, Leeds, UK; and [3]Graduate School of Agricultural Science and Biosignal Research Center, Kobe University, Kobe, Japan

ABSTRACT It is important to understand the behaviors of fluorescent molecules because, firstly, they are often utilized as probes in biophysical experiments and, secondly, they are crucial cofactors in biological processes such as photosynthesis. A phenomenon called "fluorescence quenching" occurs when fluorophores are present at high concentrations, but the mechanisms for quenching are debated. Here, we used a technique called "in-membrane electrophoresis" to generate concentration gradients of fluorophores within a supported lipid bilayer, across which quenching was expected to occur. Fluorescence lifetime imaging microscopy (FLIM) provides images where the fluorescence intensity in each pixel is correlated to fluorescence lifetime: the intensity provides information about the location and concentration of fluorophores and the lifetime reveals the occurrence of energy-dissipative processes. FLIM was used to compare the quenching behavior of three commonly used fluorophores: Texas Red (TR), nitrobenzoaxadiazole (NBD), and 4,4-difluoro-4-bora-3a,4a-diaza-*s*-indacene (BODIPY). FLIM images provided evidence of quenching in regions where the fluorophores accumulated, but the degree of quenching varied between the different fluorophores. The relationship between quenching and concentration was quantified and the "critical radius for trap formation," representing the relative quenching strength, was calculated as 2.70, 2.02, and 1.14 nm, for BODIPY, TR, and NBD, respectively. The experimental data support the theory that quenching takes place via a "transfer-to-trap" mechanism which proposes, firstly, that excitation energy is transferred between fluorophores and may reach a "trap site," resulting in immediate energy dissipation, and, secondly, that trap sites are formed in a concentration-dependent manner. Some previous work suggested that quenching occurs only when fluorophores aggregate, or form long-lived dimers, but our data and this theory argue that traps may be "statistical pairs" of fluorophores that exist only transiently. Our findings should inspire future work to assess whether these traps can be charge-transfer states, excited-state dimers, or something else.

SIGNIFICANCE This study furthers our understanding of the fundamental physical behavior of fluorescent molecules, particularly the energy-dissipating "quenching" process that occurs at high molecular concentrations. Our findings have relevance to biological photosynthesis in the demonstration that a 2D system of fluorophores distributed in lipid bilayers can act as a simplified model platform where dynamic control can be exerted over fluorophore concentrations in a way that is not possible for complex, natural systems. Our findings on "transfer-to-trap" quenching are applicable to natural pigments, such as chlorophylls. For the community of researchers using bioimaging and fluorescence assays, our findings highlight that fluorescent probes must either be used at low concentrations or quenching effects must considered (and corrected for) if concentrations are high.

INTRODUCTION

Fluorescence is a fundamental physical process that occurs after a molecule absorbs light, enters a higher-energy excited state, and then releases that energy as photon emission (1). A great variety of fluorescent molecules (fluorophores) exist, ranging from synthetic organic compounds (2) to natural biomolecules (3), and they are often used as a labeling tool in biophysics research. Despite the wide-ranging use of fluorophores, their behavior is not fully understood, particularly the process of "quenching" that can occur when multiple fluorophores interact together (4). Concentration-dependent self-quenching is a phenomenon observed in experiments where the fluorescence

Submitted February 22, 2024, and accepted for publication July 19, 2024.
*Correspondence: p.g.adams@leeds.ac.uk
Editor: Manuel Jose Estevez Prieto.
https://doi.org/10.1016/j.bpj.2024.07.026
© 2024 The Authors. Published by Elsevier Inc. on behalf of Biophysical Society.
This is an open access article under the CC BY license (http://creativecommons.org/licenses/by/4.0/).

intensity measured per fluorophore decreases nonlinearly as the concentration of fluorophores increases (5–10). In biophysical measurements, unexpected quenching can be a problem if it disrupts fluorescence assays (11–13). In the biological process of photosynthesis, the quenching of excited states plays an important role within light-harvesting (LH) pigment-protein complexes (14,15). These LH complexes perform a balancing act: they contain very high concentration of chlorophyll pigments (~250 mM) but they avoid energy loss and quenching under normal conditions (14). Interestingly, certain LH proteins switch from an active state to a highly quenched state when the biological organism is exposed to high-intensity sunlight, as a means to safely dissipate excess energy, and there is debate about the protein structural changes and photophysical pathways involved (16–25). Based on the need to understand both synthetic fluorophores and natural LH proteins (26–28), it is of fundamental importance to understand the physics behind fluorescence quenching.

Biological LH proteins have a relatively high level of complexity, containing tens of pigments of a variety of types within a relatively large system, and this can make modeling energetic transitions challenging (18,29,30). Therefore, it is instructive to perform measurements on simpler model systems where there is a single type of fluorophore in a random distribution. Previous experimental studies have assessed the extent of quenching occurring for model systems such as pigments dissolved in organic solvents (a 3D system) and pigments within a lipid membrane (a 2D system). For example, investigations of chlorophylls (5,6) and synthetic organic fluorophores (8–10) found that the onset of quenching typically occurs when the concentration of pigments exceeds ~5 mM leading to average inter-pigment distances <5 nm. Previous theoretical studies have attempted to test the structural basis of concentration-based quenching by considering networks of fluorophores, developing mathematical models (9,31–35), and performing computational simulations (34,36–39). Based on the findings of both experiment and theory, there are two types of energy "traps" proposed to cause quenching in simple systems involving a single type of fluorophore: 1) ground-state dimers of fluorophores that are relatively long lived due to strong chemical or physical attractions (10,40–42), and 2) "statistical pairs" of fluorophores that do not have any attractive interactions in their ground states and only interact transiently when one of the pair of fluorophores enters an excited state (7,32,43). It is logical that ground-state aggregates of a fluorophore would lead to quenching because direct inter-molecular contact could lead to efficient energy dissipation via electron transfer processes (44), but such aggregation would require strong attractive interactions, which seem unlikely to occur for all types of fluorophores in random distributions. To better understand the behavior of fluorophores, it is important to determine which of these quenching mechanisms is the best representation in each case.

For experimental investigations of fluorescence quenching, a controllable system of fluorophores is required where the inter-fluorophore distances can be varied. The results can then be more accurately compared to the theoretical models (27). In past studies, a series of fluorophore samples across a concentration range would be prepared, for example 0.01–10 mM chlorophyll in organic solvents (5), and analyzed with fluorescence spectroscopy. Each individual sample resulted in a single datapoint on a quenching-versus-concentration graph, so it was time consuming to assess complex trends. We recently published a new method for preparing and analyzing a range of fluorophore concentrations within a single sample to allow for a deeper study of fluorescence quenching (45). In this work, the commonly used fluorophore Texas Red (TR) was incorporated within supported lipid bilayers (SLBs) at a relatively low concentration and then an electric field (E-field) was applied to generate a concentration gradient of TR. The novelty here was, firstly, to use fluorescence lifetime imaging microscopy (FLIM) to take images where the fluorescence lifetime was correlated to the fluorescence intensity of TR, allowing quenching to be quantified (45), and, secondly, to exploit the "in-membrane electrophoresis" technique (46–51) to study quenching. We found that electrophoresis generated a roughly exponential gradient of TR concentration across an SLB and that concentrations of >2% TR (mol/mol % relative to total lipid) were correlated to significant quenching (45). A knowledge gap highlighted by this work was that the molecular basis for the energy dissipation underlying quenching was unknown. Furthermore, only one type of fluorophore was studied and we noted that different types of fluorophores could have different quenching behaviors due to different chemical structures and physical characteristics.

In this paper, we use in-membrane electrophoresis and FLIM to compare three different organic fluorophores, allowing us to contrast the quenching behavior of different fluorophore chemistries. To assess the mechanism of quenching, our experimental results are compared with a theoretical model for "transfer-to-trap" quenching.

MATERIALS AND METHODS

Theory

Theoretical model for fluorophore quenching as a function of concentration in 2D

At its simplest level, our experimental model system produces a defined quantity of fluorophores in a random arrangement within a 2D plane. One benefit of localizing fluorophores to a very thin film is that reabsorption (inner filter) effects, which distort the spectra of concentrated 3D solutions, are avoided (35). This model system is a self-assembled 2D film of lipids containing certain quantities of fluorescent probes (Fig. 1 *A*),

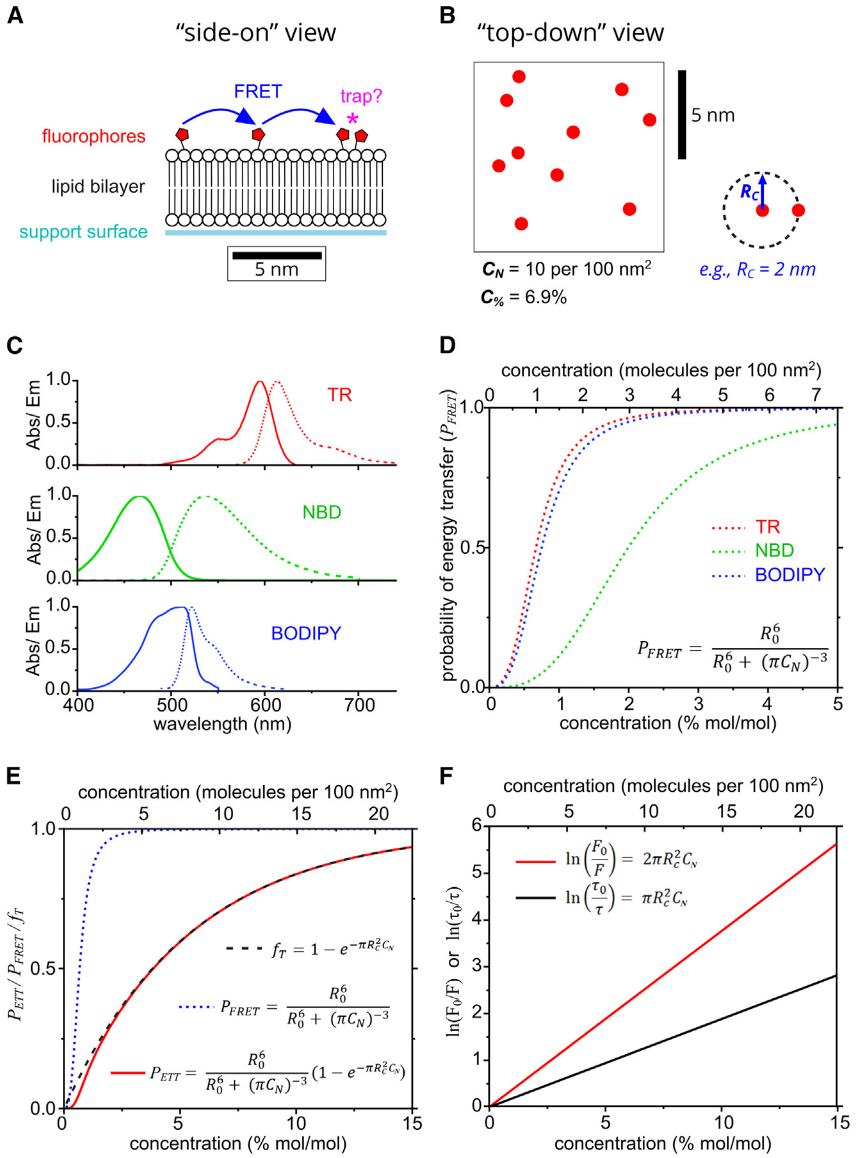

FIGURE 1 Schematics and calculations of energy transfer and trapping properties for TR, NBD, and BODIPY based on theoretical models. (*A*) Cartoon of the model membrane system containing a small number of fluorophores tethered to lipid headgroups, drawn to scale (viewed from a side-on perspective). A hypothetical trap site formed by a statistical pair is denoted by a pink asterisk. (*B*) A top-down view of a 10 × 10-nm box containing 10 fluorophores, at the same scale as (*A*). This represents a number density of $C_N = 0.1$ nm^{-2}, equivalent to $C_\% = 6.9\%$ mol/mol. The fluorophore is assumed to occupy the same area as a lipid (0.69 nm^2). (*C*) Absorption and fluorescence spectra of TR, NBD, and BODIPY (as labeled) used to calculate the Förster radius of each fluorophore. (*D*) Theoretical plots of the probability of FRET between two fluorophores as a function of their concentration, according to the equation shown. (*E*) Theoretical plots of P_{FRET}, f_T, and P_{ETT} for TR using $R_0 = 5.71$ nm and $R_C = 2.00$ nm (as labeled). A schematic depicting $R_C = 2$ nm is shown in (*B*). (*F*) Theoretical plots of the logarithm of the relative fluorescence intensity or lifetime as a function of fluorophore concentration, as expected for quenching due to the transfer-to-trap model.

but the same theory will apply to other 2D systems. For simplicity, we consider the energy transfer within only one leaflet of the lipid bilayer and assume that both leaflets have the same distribution of fluorophores. To identify the molecular mechanism of fluorophore self-quenching, we apply mathematical models that were previously suggested to represent quenching effects and adapt them for our experimental system. The majority of equations have been derived previously (as cited), and here they are applied to predict quenching relationships for our fluorophores of interest and to derive an expression for analysis of FLIM data (Eq. 23).

First, the distances at which interactions between fluorophores could take place were considered. Previous microscopy measurements of lipid bilayers containing a range of fluorophore concentrations have shown that significant fluorescence quenching occurs at a concentration of 1% fluorophores, where % represents the percentage of total lipid molecules that are fluorescently tagged (45). The fluorophore concentration can be converted from this mole-to-mole percentage, as typically reported in experiments, to a number density because this is more useful for spatial considerations:

$$C_N = \frac{C_\%}{100} \times \frac{1}{A_{lip}} \tag{1}$$

where C_N and $C_\%$ are the concentration of fluorophores in molecules per nm^2 (i.e., number density) and in mol/mol %, respectively, and A_{lip} is the area occupied by a single lipid (estimated to be 0.69 nm^2 (52)). The cartoon in Fig. 1 *B* shows an example C_N of 0.1 per nm^2 equivalent to $C_\%$ ~6.9%.

Next, the average distance between molecules (r, center to center), can be related to their concentration via the area of a circle drawn around the molecule:

$$\text{Effective area per molecule} = \frac{1}{C_N} = \pi r^2 \tag{2}$$

Therefore:

$$r = \pi^{-\frac{1}{2}} C_N^{-\frac{1}{2}} \tag{3}$$

Thus, for a membrane containing a typical concentration of 1% mol/mol fluorescent lipids, the average distance between fluorophores can be calculated as 4.69 nm. Other expressions for the average distance between molecules were also evaluated, such as directly considering a statistical distribution of nearest neighbors, but the mathematical difference was minimal between Eq. 3 and the alternative (see derivation and Figs. S1 and S2 in the supporting material).

Now that concentrations and distances have been considered, a theoretical model for concentration quenching can be applied. One possibility is that an excited fluorophore may diffuse into direct contact with a nearby fluorophore whereby the fluorescence is quenched by the collision of the molecules, sometimes termed "collisional quenching." To test this possibility, the properties of the TR fluorophore were used to calculate the mean displacement due to diffusion, $<x>$, over a time equal to the fluorescence lifetime, as a representation of the period that an excited state would typically persist:

$$<x> = \sqrt{4D\tau_0} \tag{4}$$

where D is the diffusion constant ($D = 2.27\ \mu m^2/s$, Fig. S3) and τ_0 is the fluorescence lifetime for TR ($\tau_0 = 4.23$ ns; Table S3). $<x>$ was found to be $\sim$0.20 nm, significantly lower than the fluorophore separation distance of 4.69 nm, clearly suggesting that molecular collisions would occur infrequently in this system, so this cannot explain the significant quenching (45). Thus, we must consider models that involve the transfer of excited states over a distance of several nanometers. The most likely means is the nonradiative transfer of energy via resonance interactions as described by Förster theory (53), whereby an individual excited state can "hop" between fluorophores.

Numerous studies have proposed that self-quenching processes involve a combination of two important processes: excited-state transfer and energy dissipation. The overarching idea is that an exciton quasiparticle may rapidly migrate between multiple fluorophores and finally become quenched at a "trap site" (Fig. 1 *A*) (9,32). This will affect the observed fluorescence intensity and lifetime, as follows.

(1) Two fluorophores that are closer than a critical distance will form a trap site. Traps are nonfluorescent by definition (either photon absorption is forbidden or nonradiative dissipation of energy is immediate). Thus, the overall fluorescence intensity is reduced by the fraction of fluorophores involved in trap sites, as follows:

$$\frac{F}{F_0} = 1 - f_T \tag{5}$$

where F_0 is the original fluorescence intensity, F is the fluorescence intensity after this type of quenching, and f_T is the fraction of fluorophores involved in traps. This is sometimes termed "static quenching."

(2) In addition, fluorophores that are not part of traps may become excited and then transfer the exciton to other fluorophores via Förster resonance energy transfer (FRET). If FRET causes an exciton to reach one of the trap sites described in 1) then the energy is immediately dissipated. In this process, the transfer mechanism provides a route for quenching to occur over relatively long distances (several nanometers) and the trap provides a process for energy dissipation. This dissipative pathway is an alternative to fluorescence, resulting in a reduction in both the fluorescence intensity and fluorescence lifetime. This is termed transfer-to-trap quenching. The relative change in lifetime should be equal to:

$$\frac{\tau}{\tau_0} = 1 - P_{ETT} \tag{6}$$

where τ_0 is the original fluorescence lifetime, τ is the fluorescence lifetime after quenching, and P_{ETT} is defined as the probability of excitation transfer to a trap site.

The fluorescence intensity will also be reduced in the same manner:

$$\frac{F}{F_0} = 1 - P_{ETT} \tag{7}$$

These two processes will occur simultaneously in a system that undergoes concentration-dependent quenching, so that the fluorescence intensity is reduced by 1) and 2), whereas the lifetime is only affected by 2). So, the overall change in fluorescence intensity will be represented by the combination of both processes and the complete expression is the combination of Eqs. 5 and 7:

$$\frac{F}{F_0} = (1 - f_T)(1 - P_{ETT}) \tag{8}$$

Now, the physical origin of f_T and P_{ETT} must be considered. Our theoretical model will consider a random distribution of molecules that represent the situation of traps being statistical pairs of fluorophores. If this minimal model is sufficient to explain experimental data on fluorescence quenching, then it would imply that concentration quenching does not require molecular aggregation. For a random distribution of particles in a 2D plane with a concentration, C, the number of particles separated by a distance less than R can be derived statistically (32,54). In a quenching model system, this approach can be used to determine the fraction of fluorophores that are part of traps, f_T, as:

$$f_T = 1 - e^{-\pi R_C^2 C_N} \tag{9}$$

$$f_T = 1 - e^{-C_N/C_C} \tag{10}$$

where R_C is a property of the fluorophore known as the "critical radius for trap formation," which represents the propensity of two fluorophores to form a trap site when in close proximity (7,32). R_C is defined as the distance at which $f_T = (1 - e^{-1}) \approx 0.63$, and this leads to an equivalent "critical concentration," C_C (i.e., at $C_N = C_C$ the $f_T \approx 0.63$). Therefore, a fluorophore that has a higher R_C has the ability to form traps more readily and will self-quench more strongly. The R_C value calculated has the implicit assumption that both the transfer-to-trap and the energy dissipation process at a trap site is relatively rapid; i.e., there should be no significant change in the positions of the excited fluorophores during the quenching process. Although the energy dissipation mechanism is debated, all of the proposed quenching mechanisms proceed on a sub-nanosecond timescale (trapping by charge-transfer states or dark states, see "discussion" section). The process of transfer to trap by resonance energy transfer will occur on the nanosecond timescale. Therefore, these traps can be considered as quasi-stable and immobile for the duration of an excited state due to the slow lateral ($<x> \sim 0.2$ nm) and rotational diffusion of fluorophores compared to their fluorescence lifetimes.

The probability that any given exciton reaches a trap site can then be expressed as:

$$P_{ETT} = P_{FRET} \times f_T \tag{11}$$

where P_{FRET} is the probability of resonance energy transfer, as described by Förster theory (53), and f_T is the fraction of fluorophores that exist as traps as defined earlier. This expression assumes that an excited molecule only undergoes FRET to its nearest-neighbor, justified by multiple studies (33,36). To assess whether transfer-to-trap quenching could be a good model for our system, graphs of the theoretical P_{FRET}, f_T, and P_{ETT} were calculated as a function of the concentration of fluorophores that can be studied experimentally, as described below.

P_{FRET} depends upon the strength of dipole-dipole coupling between a potential donor and acceptor molecular, and this has a strong distance dependence. The potential for dipole-dipole coupling (and also energetic coupling) can be encapsulated in the expression for Förster radius, R_0 (53):

$$R_0^6 = 8.79 \times 10^{-5} J \kappa^2 n^{-4} \phi \tag{12}$$

where κ represents the relative orientation of the donor and acceptor transition dipoles, n is the optical refractive index of the medium, ϕ is the fluorescence quantum yield of the donor, and J is the spectral overlap integral

between the donor emission and acceptor absorption. In other words, R_0 quantifies the potential of a donor-acceptor pair to transfer excitation energy where a higher value leads to greater FRET. R_0 is defined as the inter-fluorophore separation distance at which $P_{FRET} = 50\%$.

R_0 was calculated for three different fluorescent probes that were used in our experiments with lipid bilayers: TR, nitrobenzoaxadiazole (NBD), and 4,4-difluoro-4-bora-3a,4a-diaza-*s*-indacene (BODIPY). In our scenario where the exciton donor and acceptor are identical molecules, a value for J can be calculated as the area overlap between the measured emission and absorption spectra for each fluorophore (9,10), as shown in Fig. 1 *C*. Graphical integration of these overlaps found values of 3.08, 0.28, and 2.13×10^{15} $M^{-1}cm^{-1}nm^4$ for TR, NBD, and BODIPY, respectively. For our system, the fluorescent moiety was assumed to be randomly orientated due to the effects of both lateral diffusion of the lipids and rotational diffusion of the tethered fluorophores, equating to a value for κ^2 of 2/3 (55). We did also consider the possibility that fluorophores have some preferred orientation and concluded that, firstly, this was unlikely, and, secondly, even if it did occur, it would have a limited effect on quenching anyway (see justification in section "Consideration of the relative orientation of fluorophores" in the supporting material). The refractive index of the optical medium was given a value of 1.45, halfway between that of water (1.33) and lipids (1.55), as in previous estimates (56). The fluorescence quantum yield of TR, NBD, and BODIPY has been previously determined as 0.93, 0.40, and 0.99 (9,57,58). Using these values and Eq. 12, the Förster radius R_0 was found to be 5.73, 3.32, and 5.41 nm for TR, NBD, and BODIPY, respectively. Therefore, the potential for FRET is roughly similar for TR and BODIPY and lower for NBD.

Next, P_{FRET} was calculated as a function of concentration for the three fluorophores using the values determined for R_0 and a range of concentrations, C_N, and the following expression from Förster theory:

$$P_{FRET} = \frac{R_0^6}{R_0^6 + r^6} = \frac{R_0^6}{R_0^6 + (\pi C_N)^{-3}} \tag{13}$$

The results of these calculations are plotted in Fig. 1 *D*, where it can be seen that, for all fluorophores, the probability of FRET increases rapidly as a function of concentration before approaching unity at $C_{\%}$ ~2% for TR and BODIPY and ~5% for NBD. This result shows that, for the typical concentrations of fluorophores found in lipid bilayers, excitation energy transfers are highly likely to occur, leading to efficient random migration of excitons throughout the membrane.

Now, starting from the definition in Eq. 11, P_{ETT} can be re-written by combining Eqs. 13 and 9 as:

$$P_{ETT} = \frac{R_0^6}{R_0^6 + (\pi C_N)^{-3}}\left(1 - e^{-\pi R_C^2 C_N}\right) \tag{14}$$

It is important to note that this theoretical quenching probability (P_{ETT}) is equivalent to the experimentally observable quenching behavior:

$$P_{ETT} \equiv QE \tag{15}$$

where QE is the "quenching efficiency" calculated from experimental measurements of fluorescence lifetime (i.e., spectroscopy), as follows:

$$QE = 1 - \frac{\tau}{\tau_0} \tag{16}$$

To generate graphs of the theoretical quenching behavior, the values for f_T, P_{FRET}, and P_{ETT} (Eqs. 9, 13, and 14, respectively) were calculated for a range of TR concentrations. These theoretical calculations used the value for R_0 (TR-TR) = 5.73 nm (as determined above) and a placeholder value for critical radius of trap formation, $R_C = 2.0$ nm, and inputted a range of $C_{\%} = 0\%$–15%. This value for R_C is a reasonable first estimate, based on previous reports on other fluorophores (9,10), but this parameter will be quantified experimentally later in this report. Fig. 1 *E* shows the theoretical f_T (black), P_{FRET} (blue), and P_{ETT} (red) versus concentration curves for TR. All three curves tend asymptotically toward unity, but the rate of this approach varies drastically. P_{FRET} increases steeply with concentration before asymptotically tending toward unity at concentrations above ~2%, whereas f_T and P_{ETT} increase with shallower gradients and only begin to tend toward unity at much higher concentrations (over 15%). Most significantly, the theoretical plots demonstrate the similarity between the P_{ETT} and f_T curves, which are almost perfectly overlaid for the concentrations of 1.5%–15%. At lower concentrations, P_{ETT} deviates from f_T and at ~1% TR there is a difference of ~25%. This suggests that transfer-to-trap quenching should be dominated by the number of traps in the membrane for TR and that, at sufficiently high fluorophore concentrations of >1.5% TR, it is reasonable to assume that $P_{ETT} \approx f_T$ (i.e., that $P_{FRET} \approx 1$). The multiple redundant pathways for FRET between fluorophores toward a trap site increase the overall efficiency and make this assumption even more reasonable (i.e., multiple options for exciton hopping where several fluorophores are nearby). Furthermore, energy transfer could occur between fluorophores in different leaflets of the lipid bilayer, given a membrane width of ~4 nm, and this would further increase P_{FRET}.

Having established the potential of these theoretical models, we wished to apply them to our experimental observations of fluorescence to gain a better understanding of the quenching mechanism. Using the approximation that $P_{ETT} = f_T$, simplified relationships can be derived that relate a reduction in fluorescence lifetime and intensity to these statistical models for quenching (9).

Firstly, considering that the fluorescence lifetime is affected by transfer-to-trap quenching (and not static quenching), starting with Eq. 6, and substituting in $P_{ETT} = f_T$ and Eq. 9, the relative change in lifetime is:

$$\frac{\tau}{\tau_0} = e^{-\pi R_C^2 C_N} \tag{17}$$

This can be written in a semi-logarithmic format to provide a linear relationship between fluorophore concentration and the amount of quenching:

$$ln\left(\frac{\tau_0}{\tau}\right) = \pi R_C^2 C_N \tag{18}$$

or, alternatively, the change in lifetime can be related to QE (combining Eqs. 16 and 17):

$$QE = 1 - e^{-\pi R_C^2 C_N} \tag{19}$$

Secondly, considering that the fluorescence intensity is affected by both transfer-to-trap quenching and static quenching as in Eq. 8 and then simplifying with the $P_{ETT} = f_T$ assumption:

$$\frac{F}{F_0} = (1 - f_T)^2 \tag{20}$$

Therefore (using Eq. 9),

$$\frac{F}{F_0} = e^{-2\pi R_C^2 C_N} \tag{21}$$

and, again, by rearranging and taking the natural logarithm:

$$ln\left(\frac{F_0}{F}\right) = 2\pi R_C^2 C_N \tag{22}$$

Plots of $ln(\tau_0/\tau)$ (Eq. 18) and $ln(F_0/F)$ (Eq. 22) as a function of concentration, across a typical range, are shown in Fig. 1 *F*. The linear relationships, expected for fluorescence quenching, will later be compared to experimental results of in-membrane electrophoresis to determine whether

transfer-to-trap quenching occurs for lipid bilayers containing either TR, NBD, or BODIPY. Furthermore, the gradient determined from a plot of $ln(\tau_0/\tau)$ vs. C will be used to determine the critical radius for trap formation, R_C, to quantify the quenching strength of all three fluorophores.

Finally, we can derive an equation for the expected "nonquenched" fluorescence intensity (F_0) in terms of the observed fluorescence intensity (F) and the fluorescence lifetime ratio τ/τ_0 by combining Eqs. 18 and 22 and rearranging for F_0:

$$F_0 = F \cdot e^{\left[2 \ln\left(\frac{\tau_0}{\tau}\right)\right]} \tag{23}$$

The derivations and calculations in this section show how the mechanism of transfer-to-trap quenching can be theoretically modeled for the three fluorophores studied and provide expressions for estimating the "critical radius of trap formation." In the following sections, these mathematical models will be applied to experimental measurements of fluorescence intensity and lifetimes to judge the quenching strength and mechanism for each fluorophore.

Experimental methods

Preparation of membrane corrals

1,2-Bis(10,12-tricosadiynoyl)-*sn*-glycero-3-phosphocholine (Diyne-PC) lipids and 1,2-dioleoyl-*sn*-glycero-3-phosphocholine (DOPC) lipids were purchased as solids from Avanti Polar Lipids. The fluorescently tagged lipids TR 1,2-dihexadecanoyl-*sn*-glycero-3-phosphoethanolamine (TR-DHPE), 1,2-dipalmitoyl-*sn*-glycero-3-phosphoethanolamine-*N*-(7-nitro-2-1,3-benzoxadiazol-4-yl) (NBD-DHPE), and *N*-(4,4-difluoro-5,7-dimethyl-4-bora-3a,4a-diaza-*s*-indacene-3-propionyl)-1,2-dihexadecanoyl-*sn*-glycero-3-phosphoethanolamine (BODIPY FL DHPE) were purchased as solids from Invitrogen (Thermo Fischer Scientific), Avanti Polar Lipids, and Invitrogen, respectively. Template patterns were prepared as described in previous publications (45,59,60). Briefly, SLBs of Diyne-PC were formed on glass substrates by vesicle spreading and then polymerization was conducted by UV irradiation through a photomask of the desired pattern (here, a 2D array of 100 × 100-μm boxes). Nonpolymerized Diyne-PC molecules were removed with a detergent solution (0.1 M SDS), forming empty corrals surrounded by polymerized lipid bilayers. Lipid vesicles composed of the specified ratio of fluorescent lipids to DOPC lipids were generated with standard probe sonication procedures in pure water. To form membrane corrals, a suspension of lipid vesicles (at a concentration of ~0.5 mg/mL total lipid) was incubated with a template pattern for 20 min and then rinsed with a low-ionic-strength buffer (purified water, adjusted to pH 7.5 using <0.1 mM HCl).

In-membrane electrophoresis

A custom-built electrophoresis chamber (50,51) was used to hold a glass substrate under aqueous buffer in a suitable position for microscopy and allow the application of a controlled E-field. The chamber was connected to a peristaltic pump via liquid outlets and a continuous 0.25 mL/min flow of buffer was provided during electrophoresis experiments to prevent the buildup of bubbles at the electrodes. Electrophoresis was performed on patterned membranes by applying an E-field (in-plane with the SLB) of 45 V/cm and monitored using a voltmeter throughout all experiments.

FLIM

FLIM was performed using a Microtime 200 time-resolved fluorescence microscope (PicoQuant). This system used an Olympus IX73 inverted optical microscope as a sample holder with light passing into and exiting various filter units for laser scanning, emission detection, and timing electronics. An excitation laser (561 or 485 nm) was driven in pulsed mode by a PDL 828 Sepia II burst generator module at a repetition rate of 10 MHz (pulse width 70–100 ps). An appropriate dichroic mirror and bandpass emission filter was used to select the detection range. For the TR fluorophore, the excitation was at 561 nm and the collection between 590 and 650 nm, whereas, for NBD and BODIPY, the excitation was at 485 nm and the collection between 500 and 540 nm. The detector was a hybrid photomultiplier tube and the instrument response function was measured to have full width at half maximum of 100–120 ps. An excitation fluence of 0.012 mJ cm^{-2} was used for all measurements, which produced sufficient fluorescence signal and limited any singlet-singlet annihilation events (see Fig. S3). Images were acquired by scanning the laser using a galvanometric (FLIMbee) scanner and accumulating many frames of the same region (1 frame = 3.2 s). A standard FLIM image was 25 frames (80 s of exposure), an optimal acquisition time that balanced the need for obtaining a strong fluorescence signal with the requirement to minimize photobleaching (45). The possibility of oxygen-dependent redox effects that may affect fluorophore photophysical properties was minimized by de-gassing all buffer solutions before use. Initial analysis of FLIM data was performed with SymPhoTime software (PicoQuant). The mean amplitude-weighted lifetime of images or pixels, $<\tau>$, was calculated by generating fluorescence decay curves from accumulated photons and modeling the curve as a multi-exponential decay function (excellent fits were achieved for all data, with chi-squared values <1.1 and low residuals). Secondary graphical analyses were performed with OriginPro software, as described previously (45). Calculated graphs representing the theoretical models were generated by applying the applicable formulae to appropriate input data in OriginPro software.

RESULTS

General approach of electrophoresis of fluorophores within supported lipid membranes

TR, NBD, and BODIPY were selected as suitable targets for study because, firstly, they are all commonly used fluorescent probes in lipid bilayers and, secondly, they have different chemical and optical properties that could result in a variable susceptibility to quenching or different mechanisms that would make for a good comparison. For example, TR absorbs in the green spectral region ($\lambda \sim 575$ nm), whereas NBD and BODIPY absorb at similar wavelengths in the blue region ($\lambda \sim 475$ nm). Furthermore, TR and NBD are hydrophilic (due to the presence of atoms with different electronegativity, O, N, S) whereas BODIPY is generally considered as hydrophobic (balance between nearby F and N). All three are available to purchase in a form where the fluorophore is covalently tethered onto the headgroup of a lipid. As shown in the chemical structures, the net charge of each fluorophore is zero (Figs. 2 *A–C*) but, when tethered to the negatively charged lipid DHPE (Fig. 2 *D*), each molecule has an overall negative charge, $q = -1e$, when immersed in an aqueous buffer at neutral pH. Photo-polymerizable lipids were used to generate template patterns consisting of empty 100 × 100-μm squares (Fig. 2 *F*). Then, a precise concentration of TR, NBD, or BODIPY was incorporated into vesicles comprised primarily from the neutral lipid DOPC (Fig. 2 *E*), and this solution of vesicles was incubated with the template to form patterned SLBs, termed membrane corrals (Fig. 2 *G*,

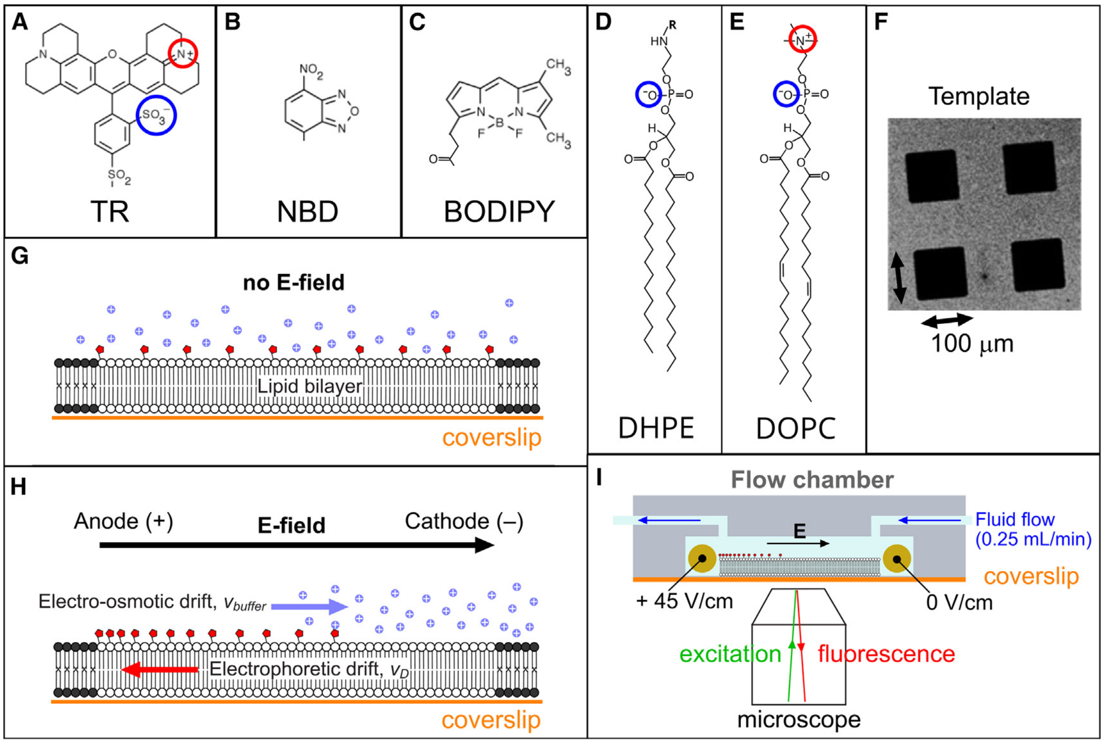

FIGURE 2 Structures and schematics of the fluorophore-lipid system. Chemical structures of the fluorophores (*A*) TR, (*B*) NBD, and (*C*) BODIPY, and (*D*) the DHPE lipid to which the fluorophores are linked (covalent attachment to replace R). (*E*) The bulk lipid DOPC. (*F*) Example fluorescence microscopy image of the template pattern of Diyne-PC lipids in a micro-array pattern, as generated by photolithography. (*G*) Schematic of a lipid bilayer confined by the barrier of photo-polymerized Diyne-PC (*black*). In the absence of any E-field, fluorophores (*red*) will be uniformly distributed in the membrane with a screen of ions (*purple*) close to the membrane surface. (*H*) As in (*G*), but with an applied E-field. (*I*) Schematic of the electrophoresis flow cell (not to scale).

typically 0.5% fluorescent lipids relative to total lipids, by weight). Fluorophores within these corrals are expected to be mobile and homogenously distributed throughout the membrane (59,60). When an E-field is applied parallel to an SLB, the negatively charged fluorophores are expected to migrate toward the positive electrode and accumulate at the impenetrable edge of the membrane corrals (Fig. 2 *H*) (59–61). The balance of forces that work for (e.g., electrophoretic force) and against (e.g., friction and electroosmotic drag) the E-field will determine the fluorophore drift velocity and, eventually, the system will reach a dynamic equilibrium where the fluorophore's concentration profile across the corral is the result of the competition between E-field-induced drift and random diffusion. To maintain the SLBs under a constant E-field and allow microscopy, samples were maintained within a custom-built flow chamber (see schematic Fig. 2 *I*). To quantify the self-quenching of each fluorophore, FLIM was used to measure the spatial distribution and corresponding fluorescent lifetimes of fluorophores during electrophoresis experiments. Initial experiments were performed on patterned SLBs containing each fluorophore to optimize the FLIM image acquisition parameters to ensure the accuracy of fluorescence data collected (accidental photobleaching and singlet-singlet annihilation were minimized, see section "Confirming the membrane quality: structural contiguity and lateral lipid diffusion" of the supporting material). Fluorescence images and 2D diffusion measurements confirmed that each fluorophore distributed homogenously throughout the membrane and had high lateral mobility (Fig. S3). These preliminary FLIM measurements of patterned SLBs confirmed that high-quality data could be acquired for all three fluorophores.

Analysis of FLIM images of fluorophores experiencing quenching

To determine the extent to which different fluorophores are susceptible to concentration quenching, electrophoresis and FLIM were used to analyze membranes containing fluorophores. Corrals of SLBs containing either TR, NBD, or BODIPY at a similar initial concentration were compared (0.5% by weight; molar concentrations were considered later) (Fig. 3). FLIM images were captured both before electrophoresis and 1 h after electrophoresis when a dynamic equilibrium had been established. Before electrophoresis, all three fluorophores had flat homogeneous distributions of fluorescence intensity across the membrane corral (Figs. 3 *A*, *E*, and *I*). Qualitatively, the effect of the E-field on the fluorescence intensity was consistent, with all three

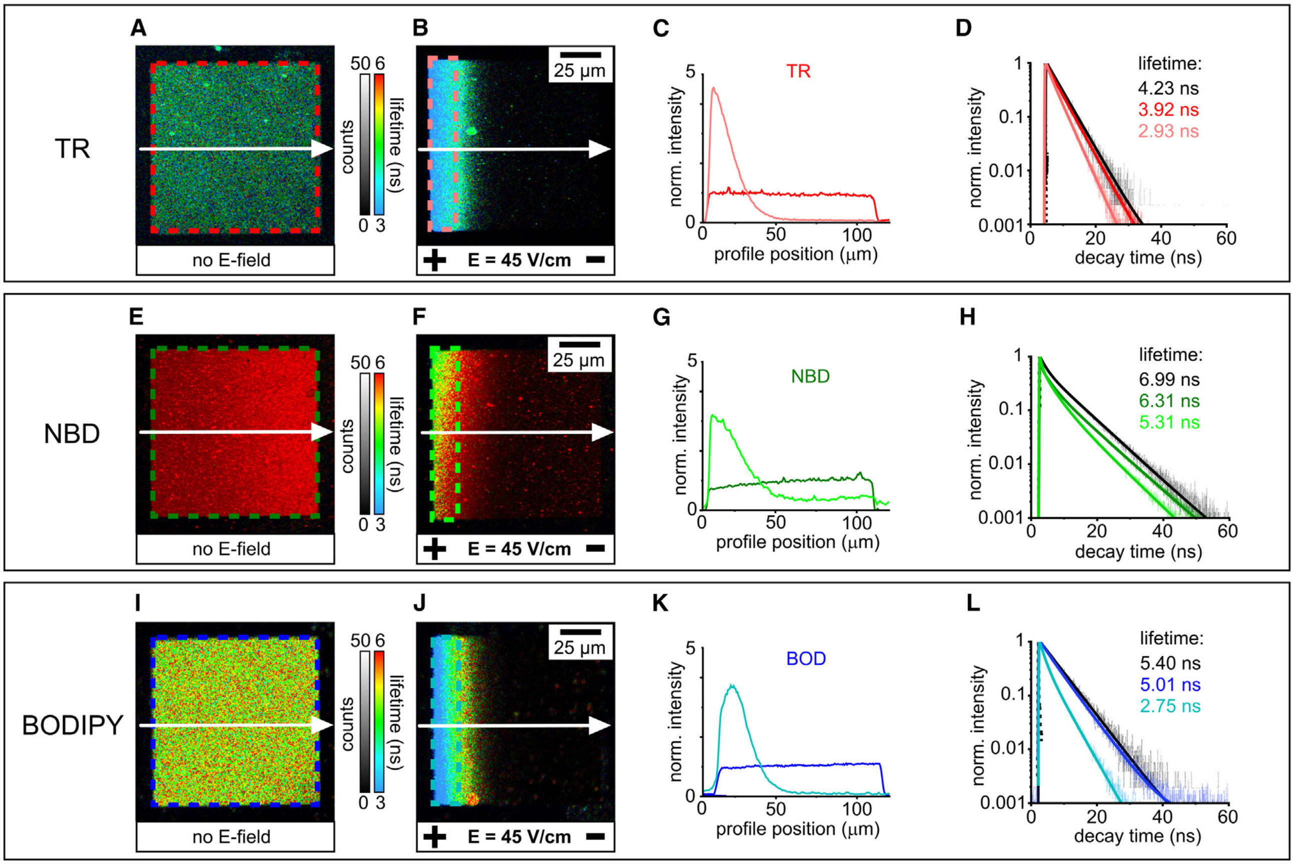

FIGURE 3 Comparison of the before- and after-electrophoresis states of membrane corrals containing TR, NBD, and BODIPY. (*A*) FLIM image of a corral containing 0.5% (w/w) TR-DHPE within a DOPC lipid bilayer before electrophoresis. The box highlighted by red-colored dashed lines is an ROI representing the entire corral. (*B*) FLIM image of the corral from (*A*) after the application of a 45-V/cm E-field for 1 h. The box highlighted by colored dashed lines is an ROI chosen to represent roughly 15% of the total corral area. This size is chosen because it is large enough to provide sufficient fluorescence signal for further analysis but small enough to select a high-concentration region where fluorophores have accumulated. (*C*) Fluorescence intensity profiles drawn across the membrane indicated by white arrows in (*A*) and (*B*), either before (*dark red*) or after electrophoresis (*light red*). Both profiles are normalized to 1.0 for the average starting intensity of the corral, to compare between samples. (*D*) Fluorescence decay curves representing the membrane corral before electrophoresis and after electrophoresis (curves colored to represent the ROI of the same color in *A* and *B*). For each, the raw fluorescence data (*thin lines*) was fit to a multi-exponential decay function (*bold lines*) and the value for mean amplitude-weighted lifetime extracted from the fit is displayed. The decay curve acquired for a control sample with even lower fluorophore concentration (0.25% TR) is shown for comparison (*black curve*). (*E–H*) FLIM images, fluorescence intensity profiles, and fluorescence decay curves of a 0.5% (w/w) NBD-DHPE lipid bilayer. (*I–L*) FLIM images, fluorescence intensity profiles, and fluorescence decay curves of a 0.5% (w/w) BODIPY-DHPE lipid bilayer.

fluorophores having an asymmetric fluorescence profile with a significantly higher intensity at the left-edge of the corral (Figs. 3 *B*, *F*, and *J*). All three fluorophores exhibited over a threefold intensity increase after electrophoresis, to 4.5× the initial intensity for TR, 3.0× for NBD, and 3.8× for BODIPY (Figs. 3 *C*, *G*, and *K*). Although not a focus of current work, the kinetics of the lipid accumulation could be tracked for each fluorophore and the electrophoretic drift velocity was very similar for all three fluorescent lipids (v_d = 0.31, 0.28, 0.27 μm/s for TR, NBD, BODIPY; see Fig. S4) and in agreement with publications on similar systems (50). The estimated concentration of fluorophores accumulated at the positive electrode is calculated later in this work (next section).

For all fluorophores, the fluorescence lifetime was significantly reduced during electrophoresis, as represented by the blue-shift in the false-color scale for lifetime (Figs. 3 *B*, *F*, and *J*). To quantify and compare the quenching between the three fluorophores, lifetimes were determined by fitting the fluorescence decay curves produced from a region of interest (ROI) at the left edge of the corral. The width chosen for these ROIs is arbitrary and simply represents an equal-sized region from each sample where the fluorophore has accumulated due to electrophoresis. It was found that the fluorescence lifetime decreases from 3.92 to 2.93 ns for TR, decreases from 6.31 to 5.31 ns for NBD, and decreases from 5.01 to 2.75 ns for BODIPY (Figs. 3 *D*, *H*, and *L*). To quantify the degree of self-quenching for each fluorophore, the *QE* was calculated from the ratiometric reduction in fluorescence lifetime (Eq. 16, see section "theory"). The *QE* observed in the ROI at the left edge of the corral at electrophoretic equilibrium was calculated as 30.7%, 24.0%,

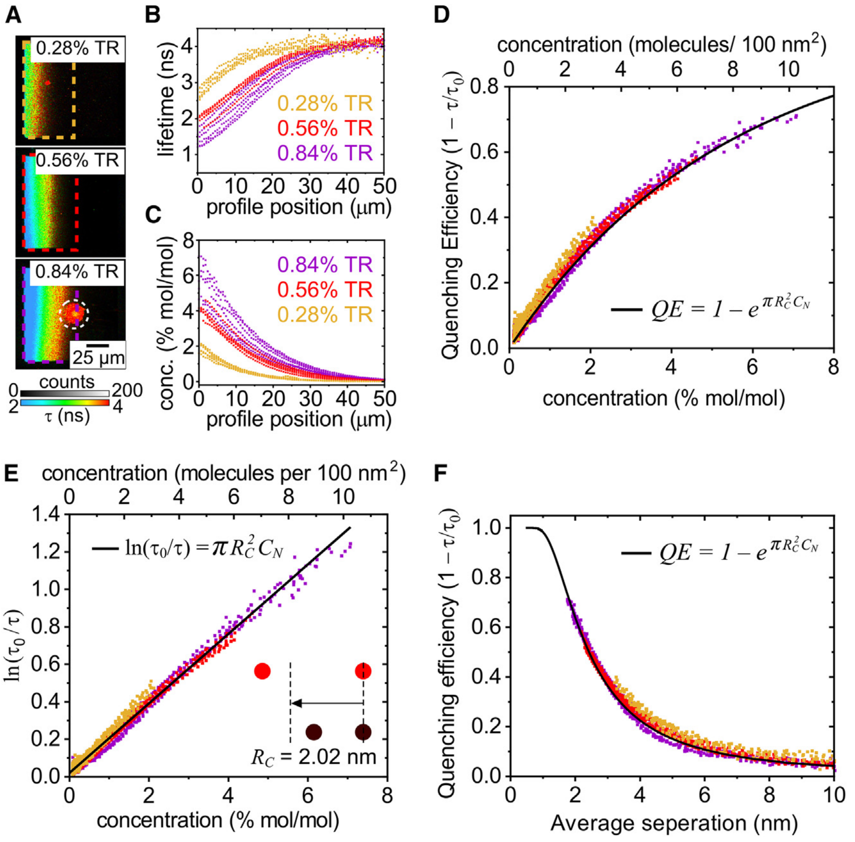

FIGURE 4 Analysis of QE against concentration for a range of TR concentrations. To account for statistical variations between different membrane corrals and improve our precision, the data from multiple corrals for each sample is accumulated ($n = 6$). (*A*) Example FLIM images of corrals containing an initial concentration of either 0.28%, 0.56%, or 0.84% (mol/mol) TR at electrophoretic equilibrium. The boxes highlighted by colored dashed lines are the ROI from which data were analyzed (see Fig. S6). The data from these ROIs were used to generate the corresponding scatter plots in (*B*)–(*F*). These ROIs were chosen because the majority of the fluorescence occurred in the left-hand portion of the corral after the electrophoretic migration of fluorophores. There was minimal fluorescence signal in the right-hand side of the corral, and this was of insufficient quality to calculate fluorescence lifetimes, so this region was ignored. The circular region indicated by white dashed lines shows an example of a region excluded from the analysis due to a defect in the membrane, likely to be a lipid tubule, as previously reported (45). (*B*) Multiple fluorescence lifetime profiles (overlaid), as obtained from many different corrals. Each profile represents the fluorescence in the left-to-right direction across one corral with averaging across the vertical direction to enhance the accuracy, as shown in Fig. S6. Yellow, red, and purple data points represent the profiles obtained from membranes containing 0.28%, 0.56%, and 0.84% TR, respectively. (*C*) Multiple calculated concentration profiles obtained from the same corrals. (*D*) QE versus concentration for TR fluorophores in SLBs. The top x axis was generated from the bottom x axis (Eq. 1). The solid black line shows the theoretical QE curve calculated using the equation shown (Eq. 19) and the value for R_C determined in (*E*). (*E*) The same data as in (*D*) plotted as the logarithm of the relative lifetime versus fluorophore concentration. A linear fit (*black line*) of the data is shown. The gradient ($m = \pi R_C^2$) of this fit was 12.8 ± 0.1 nm^2 and R_C was calculated as 2.02 ± 0.01 nm. (*F*) The same data as in (*D*) plotted as QE versus the average separation distance between fluorophores. The solid line shows the theoretical QE curve calculated as in (D).

and 49.1% for TR, NBD, and BODIPY, respectively. Overall, the results in this section show that self-quenching occurs for all three fluorophores and that quenching was induced by increasing their concentration via in-membrane electrophoresis.

Deeper analysis of *QE* vs. concentration to assess the mechanism

In previous work, we established a method to convert a fluorescence intensity profile into a concentration profile, applying it to TR fluorophores in membrane corrals (45). To do this, the fluorescence intensity is corrected for quenching by quantifying the reduction in fluorescence lifetime (using Eq. 23). The fluorophore concentration can then be obtained using the linear relationship from standard concentration curves of fluorescence intensity (see Figs. S5 and S6). Here, this method is applied first to TR, then to NBD and BODIPY, so that quenching can be directly correlated with fluorophore concentration, and then compared to theoretical models to understand the mechanism of quenching. Electrostatic repulsions between the negatively charged fluorophores may limit the final concentrations that can be achieved. Our method simply calculates the fluorophore concentrations that are observed in the system as a result of all effects, irrespective of their origin.

FLIM images of membrane corrals, fluorescence lifetime profiles, and concentration profiles for the TR fluorophore are shown in Figs. 4 *A*–*C*. The first 0–50 μm across the left-hand side of each corral contained the vast majority of the fluorophores so this region was selected as the ROI (dashed boxes in Fig. 4 *A*) for generating the fluorescence and concentration profiles (Figs. 4 *B* and *C*). The right-hand side of the corral contained a lipid bilayer depleted of fluorophores, so this region was ignored. The calculated molecular concentrations are spatially correlated to fluorescence lifetimes in the FLIM data, so these two types of data can be directly related to each other in a scatter plot to describe the quenching behavior. Fig. 4 *D* shows the resulting QE vs. C curve which combines all datasets obtained for electrophoresis of membrane corrals containing TR. The concentration is displayed as the % mol/mol of fluorophores relative to total lipids (bottom x axis, Fig. 4 *D*) and also as a number density of fluorophores per 100 nm^2 to provide a more tangible representation of concentration at sensible length

scales (top x axis, Fig. 4 *D*). From this plot we can make several observations about the quenching behavior of TR. Firstly, the *QE* vs. *C* curves obtained from samples with varying initial concentrations all fall onto one master curve, indicating a highly consistent trend for quenching behavior. Secondly, the quenching starts to occur immediately after the hypothetical zero concentration, so that, even at 0.5% TR (a typical concentration used to probe lipid bilayers), there is a *QE* of 5%–10%. Thirdly, the amount of quenching initially increases steeply with concentration before the gradient begins to decrease approaching a *QE* of one, above which it cannot increase by definition. Therefore, ~100% quenching can be expected at higher concentrations above 10% TR.

Next, these data can be compared with the theoretical relationships for transfer-to-trap quenching from the equations derived in the "theory" section (Eqs. 17 and 18). A plot of $ln(\tau_0/\tau)$ versus *C* was found to have a strongly linear fit ($R^2 = 0.98$), the predicted relationship for transfer-to-trap quenching (*black line*, Fig. 4 *E*). The gradient of this linear fit can be used to calculate the critical radius for trap formation, R_C, a measure of the overall quenching strength of a particular fluorophore that is defined as the separation distance at which two fluorophores have a characteristic (63%) likelihood to associate and form a trap. R_C for TR was calculated as 2.02 ± 0.01 nm, suggesting that two fluorophores must have very low separation distances to form a trap. For example, this is much shorter than the distance at which effective FRET occurs, roughly one-third of the Förster radius for TR (5.73 nm). So far, the quenching behavior of TR has been considered as a function of the fluorophore concentration but it is also instructive to plot the quenching behavior as a function of the distance between fluorophores. The average separation distance between fluorophores, *r*, was calculated from the fluorophore number density and *QE* was plotted against this (Fig. 4 *F*). Following the plot from right to left, the *QE* is minimal (<10%) for $r > 5$ nm, before rapidly increasing up to 70% *QE* at an average separation of ~2 nm (as expected where $r \approx R_C$). *QE* tends toward 100% at very small *r* where all of the fluorophores become trap sites (at $r \ll R_C$), as observed in the high-concentration regime of the *QE* versus *C* plot.

To assess whether the experimental data were consistent with the transfer-to-trap model, theoretical curves for *QE* were generated by using the equations describing this model (Eq. 19), inputting the desired range of fluorophore concentrations, and experimentally determined value of $R_C =$ 2.02 nm. The calculated curves of *QE* versus *C* (black line, Fig. 4 *D*) and *QE* versus *r* (black line, Fig. 4 *F*) were found to be highly consistent with the experimental datapoints for all fluorophore concentrations and equivalent separation distances. Overall, these results show that it is highly likely that TR fluorophores undergo transfer-to-trap quenching, in which fluorophores form nonfluorescent statistical pairs as a probabilistic function of concentration and excitons migrate from excited monomers to these trap sites via FRET.

Quantitative comparison of quenching behavior between TR, NBD, and BODIPY

A quantitative comparison of the quenching behavior of all three fluorophores was performed by repeating the analysis described for TR (previous section) for the NBD and BODIPY fluorophores. The *QE* vs. *C* curve generated from this analysis is shown in Fig. 5 *A*. All three fluorophores followed the same overall trend, whereby *QE* increases with *C* and the gradient of the curve becomes increasingly shallow at high concentrations, but the dependence on concentration differs. It is apparent that BODIPY (blue datapoints) self-quenches more strongly than TR (red) and NBD (green), as demonstrated by the steeper gradient of the *QE* vs. *C* curve. For example, 2% (mol/mol) of the fluorophore within a lipid bilayer led to a reduction in the fluorescence lifetime of ~10% for NBD, ~30% for TR, and ~50% for BODIPY. This implies that BODIPY fluorophores form trap sites more readily than TR or NBD. Next, to assess whether or not all three fluorophores exhibit transfer-to-trap quenching, each dataset was plotted as $ln(\tau_0/\tau)$ versus *C* and fitted to the predicted linear relationship. For all three fluorophores, the experimental data were strongly correlated to the theoretical model with a high-quality fit over all concentrations (Fig. 5 *B*), suggesting that they undergo the same quenching mechanism. To compare the relative strengths of quenching, the gradients of these linear fits were determined and R_C values were calculated as 2.70, 2.02, and 1.14 nm for BODIPY, TR, and NBD, respectively. Therefore, we find that the quenching strength of BODIPY is 1.3 times that of TR, which is 1.8 times that of NBD.

To consider how these trends relate to distances between molecules, *QE* vs. *r* plots were generated for each fluorophore (Fig. 5 *C*). As expected, following the trend lines from right to left, BODIPY begins to quench at higher separations than TR and NBD, and BODIPY reaches a *QE* of 50% at ≈3.3 nm compared to ≈2.4 nm for TR and ≈1.4 nm for NBD. At decreasing separation distances, toward zero, the QE continues to increase and may be expected to saturate at $QE = 1$ for very low separations. Theoretical quenching curves generated using the fitted values for R_C show strong correlation to the *QE* versus *C* data (solid lines, Figs. 5 *A* and *C*). Overall, the strong correlation between experimental data and the theoretical relationship representing quenching by statistical pairs (traps) and excitation energy transfer throughout the membrane (transfer to trap) is good evidence that this mechanism does indeed occur for all three fluorophores. A final consideration of the accuracy of the trends for fluorescence quenching can be made now that

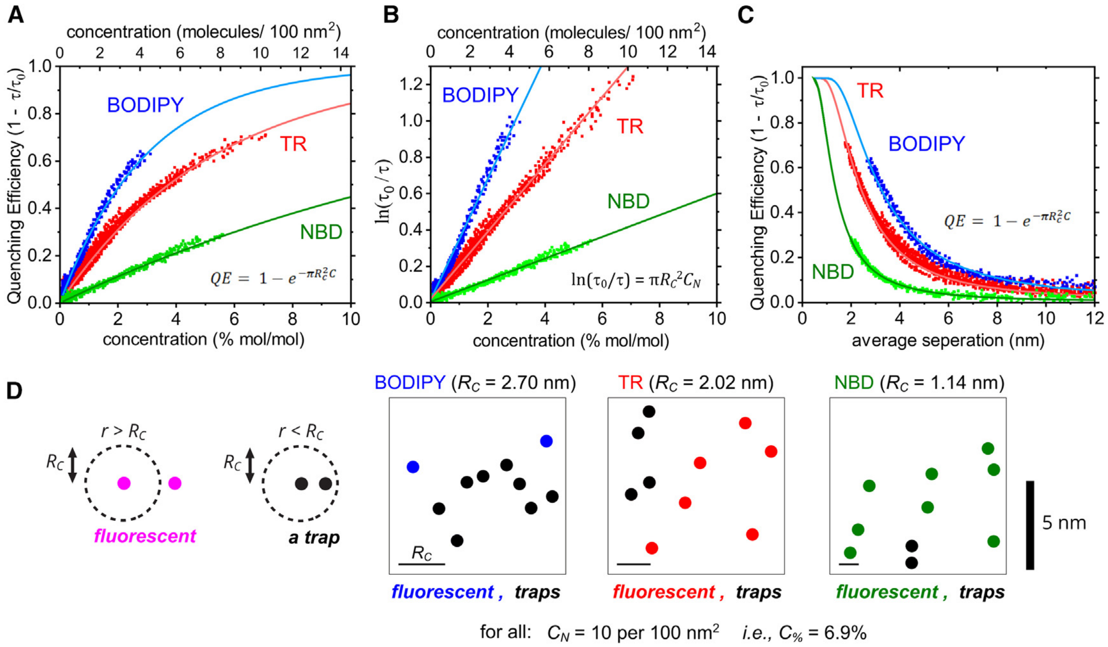

FIGURE 5 Graphs comparing the quenching relationships between TR, NBD, and BODIPY. (*A*) *QE* versus concentration for each fluorophore type. The solid lines show the theoretical *QE* curves calculated using the equation shown (Eq. 19) and the values for R_C determined in (*B*). (*B*) The same data as in (*A*), plotted as the logarithm of the relative lifetime versus fluorophore concentration. Linear fits (*solid lines*) of the equation shown were used to obtain the critical radius for trap formation, R_C. The gradient ($m = \pi R_C^2$) of each fit was 23.0 ± 0.1, 12.8 ± 0.1, and 4.11 ± 0.02 nm^{-2} for BODIPY, TR, and NBD, respectively, leading to $R_C = 2.70 \pm 0.01$, 2.02 ± 0.01, and 1.14 ± 0.01 nm. (*C*) The same data as in (*A*) plotted as *QE* versus average separation distance between fluorophores. The solid lines show the theoretical *QE* curves calculated as in (*A*). (*D*) Representations of a 10 × 10-nm region of lipid bilayer containing either TR, NBD, or BODIPY showing fluorophores that are either fluorescent (*color*) or acting as traps (*black*), according to the R_C values calculated in (*B*). This is an oversimplification because R_C is not a threshold value but instead a relative measure of the propensity to quench. Each box contains the same concentration of fluorophores, but different numbers of traps exist due to the different R_C.

R_C values have been determined for each fluorophore. A simplified mathematical model of quenching has been used so far that assumes FRET always occurs, so that the equations could be solved and R_C quantified (see "theory" section). The simplified model for quenching ($P_{FRET} = 1$) was compared with the exact model (P_{FRET} varies) by calculating the fluorescence intensity and lifetime as a function of concentration using the appropriate equations and R_C values (see Fig. S7). There was only an observable discrepancy at low fluorophore concentrations (when P_{FRET} drops), and this caused only minor differences in fluorescence intensity and lifetime. This is logical because, at low concentrations, there is little quenching anyway, and, at high concentrations, the simplified model is very close to the exact model. This confirms that quenching is dominated by the fraction of traps, rather than the FRET efficiency, for all three fluorophores.

To visualize the number of traps sites expected to exist based on the calculated critical radii, a schematic was drawn to show the same concentration of fluorophores for each fluorophore in a random distribution with trap sites indicated based on the calculated R_C values (Fig. 5 *D*). At the ∼7% concentration shown, it is evident that the majority of BODIPY molecules would exist as traps, as compared to very few traps for NBD and an intermediate number of traps for TR.

DISCUSSION

Mechanism of quenching: Transfer to trap and statistical pairs

From 1950 to 2000, there were several experimental and theoretical investigations of randomly distributed pigments as a model system for photosynthesis, particularly before the biological LH structures were known to be protein-pigment complexes. These studies proposed that the concentration-induced fluorescence quenching of chlorophyll pigments could arise either due to the occurrence of long-lived physical aggregates at very high pigment concentrations (over 100 mM) or due to statistical pairs via a transfer-to-trap mechanism at lower pigment concentrations (at 2–100 mM) (5–7,31,32,34,36,42,43,62) (see Fig. 1 and "theory" section). Exhaustive experiments by Porter et al. ruled out other energy dissipation mechanisms for chlorophyll, such as quenching by intersystem crossing to triplet states, quenching by singlet-singlet annihilation effects, or quenching by molecular collisions (6,7,34,43). Long-lived aggregates were excluded as a possible source of quenching

by the lack of changes to spectra as concentration was increased (6,7). These authors concluded that quenching must occur via a transfer-to-trap mechanism for chlorophyll. In this paper, we apply the mathematical model representing the transfer-to-trap mechanism to our experimental data to assess whether it could explain the quenching of three fluorophores that are popular probes in biophysical experiments.

Over the last decade, there has been great focus on understanding the potential mechanisms of quenching that occur in LH proteins, because quenching is thought to be crucial as part of a protective process for dissipating energy under high-intensity sunlight (15). A small number of recent studies have provided evidence for quenching by statistical pairs of chlorophylls using Monte Carlo simulations (37), molecular dynamics simulations (39), and mathematical modeling (35). However, there is little agreement on the identity of the quencher in LH proteins, possibly due to the high complexity of the system (16–25). Therefore, we decided that our newly optimized experimental technique to generate concentration gradients of fluorophores and analyze them with an advanced form of microscopy would be an ideal platform to test for agreement or disagreement with previously proposed theoretical mechanisms and, furthermore, to compare multiple fluorophores that have different chemical structures. In this paper, we quantified the fluorescence intensity and kinetics of three organic fluorophores: TR, NBD, and BODIPY. The mechanism of self-quenching for all three fluorophores was found to be a combination of the concentration-dependent formation of trap sites (statistical pairs) and the transfer of excitation energy between fluorophores until they reach these sites, i.e., transfer to trap. The evidence for this and its wider implications is highlighted below.

Fluorophores undergoing electrophoresis within lipid bilayers were observed using fluorescence microscopy, and the fluorescence lifetimes were analyzed, allowing the local concentration of fluorophores to be cross-correlated to their quenching (Figs. 3, 4, and 5). The extent of quenching for TR, NBD, and BODIPY was found to increase with fluorophore concentration before tending toward unity at very high concentrations (Fig. 5). For all three fluorophores, the mathematical expression representing the transfer-to-trap theory was in excellent agreement with the experimental data suggesting that this is the correct explanation for quenching. The important implication here is that self-quenching depends upon statistical pairs of fluorophores that do not require any interaction between their ground states (7,9,31,32). In other words, traps may occur simply due to the temporary proximity of one fluorophore to another at a given concentration and do not require chemical/physical interactions to maintain a specific molecular configuration over the long term. This may explain why such quenching behavior appears to be a general phenomenon of both natural and synthetic pigments that do not share chemical similarities and thus have no reason to self-associate in similar manners. There has been excellent work that focuses on either chlorophyll or organic fluorophores, but there is not always cross-pollination between the photosynthesis and physical chemistry communities, and we would like to highlight that the early theories developed to explain chlorophyll behavior can apply to typical fluorescent probes. Our electrophoresis method provides a direct visualization of the accumulation of molecules causing the onset of quenching, and the quantitative analysis of the data shows that this occurrence is remarkably similar for three different fluorophores. It may be possible for future researchers to direct the migration of chlorophylls within (or tethered to) lipid bilayers using electrophoresis.

Comparison of the quenching strength of three different fluorophores

Although the self-quenching of all three fluorophores could be explained by a transfer-to-trap model, there were significant differences in the extent of this quenching (Figs. 3 and 5). The critical radii for trap formation, representing the relative quenching strengths, were found to be $R_C = 2.70$, 2.02, and 1.14 nm for BODIPY, TR, and NBD, respectively. These R_C are in close agreement to those found in previous investigations of self-quenching of NBD (9) and BODIPY (10) and give us confidence in the accuracy of our analyses. To our knowledge, the R_C of TR has not previously been reported. To summarize, BODIPY undergoes concentration-dependent quenching most strongly, followed by TR at an intermediate level, and then NBD at a lower level. Previous studies reported that the R_C of chlorophyll is ≈ 1 nm (32,37) and, therefore, it is a relatively weak quencher similar to NBD. The difference in critical radii between TR, NBD, and BODIPY is likely to be due to the different physicochemical and photophysical properties of each particular chemical species. We note that the potential for trap formation does not follow the same trend as the potential for FRET represented by their Förster radii, R_0 (5.41, 5.73, and 3.32 nm for BODIPY, TR, and NBD). NBD has both the lowest R_C and R_0 so we can speculate that the relatively poor energetic coupling between an excited-state NBD and a ground-state NBD may hinder both FRET and trap formation. In contrast, BODIPY has a lower R_0 but a higher R_C than TR, so there must be something beyond the Förster coupling parameters that affects trap formation.

One possibility is that chemical differences may cause some fluorophores to have a greater potential for attractive interactions than other fluorophores and may form long lived (physical) dimers or larger aggregates. As TR, NBD, and BODIPY are negatively charged (in water at pH 7), they will experience electrostatic repulsion, but this must be balanced against any attractive interactions such as those between the nonpolar tail groups of the lipids that the fluorophores are tethered to. BODIPY has been reported to aggregate at high concentrations as a result of hydrophobic

interactions between planar moieties of the molecule (41,63). Similar face-to-face "H-type" dimers (or H-aggregates) have also been suggested to occur for chlorophylls at very high concentrations (37,42). Long-lived dimers could explain the greater quenching strength (greater R_C) of BODIPY relative to TR/NBD; i.e., there could be some combination of the transfer-to-trap model and the aggregate model for BODIPY. Absorbance spectroscopy could identify (or rule out) the presence of long-lived physical dimers/aggregates (42,64); however, absorption spectroscopy of SLBs on glass coverslips was not possible with our instrumentation. Although our experiments do rule out the presence of ground-state dimers, we highlight that the transfer-to-trap theory with statistical pairs was sufficient to model the data.

The molecular identity of the trap site and the energy dissipation pathway

The transfer-to-trap model defines the relationship between fluorophore concentration and their tendency to form trap sites; however, it does not specify the photophysical mechanism for quenching (i.e., how energy is dissipated once an excited state reaches a trap site). Two photophysical mechanisms of energy dissipation have been proposed in previous studies of quenching of fluorophore pairs. The first mechanism is quenching by a "dark state," where the trap site is a pair of fluorophores that undergo "excitonic splitting" to form one super-radiant state and one dark state (37,65,66). Fluorescence is disallowed from the dark state, so nonradiative decay will occur to dissipate the energy as heat. Such split excitonic states have been suggested to occur for H-aggregates and excimers of fluorophores (37,41,63,67). Alternatively, a second mechanism is quenching via a charge-transfer state, sometimes called photoinduced electron transfer. In this mechanism, the trap site is a pair of fluorophores that form a charge-transfer (ion-pair) state after receiving excitation energy (39,40,62). This charge-transfer state undergoes rapid recombination to regenerate the ground state and, in the process, dissipate the energy as heat (43,44). Both of these proposed mechanisms have a unique spectral signature; however, this is technically challenging to observe experimentally because of the transient nature of the molecular species. Although the molecular identity of the quencher was not investigated in the current study, we would like to highlight that it would be an interesting topic for future studies. Transient absorption spectroscopy could resolve changes in the nature of the excited state at the very short timescales involved (21,68,69), and Stark fluorescence spectroscopy can identify charge-transfer states (69,70). Completing our understanding of both the occurrence of the traps and their molecular identity will result in a more holistic understanding of how concentration-induced fluorescence quenching can occur in model systems and would have direct relevance as potential mechanisms of quenching in biological LH pigment-protein complexes. Future studies could use the electrophoresis/FLIM approach as a novel method to generate high concentrations of LH proteins and quantify the resultant quenching.

DATA AND CODE AVAILABILITY

All relevant raw and analyzed data associated with this paper are openly available under a CC-BY license in the Research Data Leeds repository (71) and can be found at https://doi.org/10.5518/1480.

SUPPORTING MATERIAL

Supporting material can be found online at https://doi.org/10.1016/j.bpj.2024.07.026.

AUTHOR CONTRIBUTIONS

P.G.A. and S.A.M. conceptualized the project. S.A.M. developed theory, prepared samples, and performed FLIM analyses. Y.K. prepared the template patterns for microscopy. P.G.A., S.D.E., S.D.C., and K.M. provided supervision over the course of the project and acquired funding. S.A.M. and P.G.A. wrote the first draft of the manuscript. All authors contributed to revising the manuscript.

ACKNOWLEDGMENTS

S.A.M. was supported by a PhD studentship from the Biotechnology and Biological Sciences Research Council UK (BBSRC) (BB/M011151/1). Y.K. and K.M. were supported by the Japan-UK Research Cooperative Program (JPJSBP120195707) and a Grant-in-Aid for Scientific research (Kakenhi) (nos. 19H04725 and 21KK0088) from Japan Society for the Promotion of Science (JSPS). S.D.E. was supported by the National Institute for Health Research infrastructure at Leeds and by the EPSRC (EP/W033151/1). S.D.C. was supported by grants from the Engineering and Physical Sciences Research Council UK (EPSRC) (EP/R043337/1, EP/R03608X/1, and EP/J017566/1). P.G.A. was supported by a University Academic Fellowship from the University of Leeds and a grant from the EPSRC (EP/T013958/1). The PicoQuant FLIM instrument at Leeds was acquired with funding from the BBSRC (BB/R000174/1).

DECLARATION OF INTERESTS

The authors declare that they have no competing interests.

SUPPORTING CITATIONS

References (72–76) appear in the supporting material.

REFERENCES

1. Lakowicz, J. R. 2006. Principles of Fluorescence Spectroscopy. Springer, New York.
2. Keerthana, S., B. Sam, ..., A. Varghese. 2021. Fluorescein based fluorescence sensors for the selective sensing of various analytes. *J. Fluoresc.* 31:1251–1276.

3. Remington, S. J. 2011. Green fluorescent protein: A perspective. *Protein Sci.* 20:1509–1519.
4. Gehlen, M. H. 2020. The centenary of the Stern-Volmer equation of fluorescence quenching: From the single line plot to the SV quenching map. *J. Photochem. Photobiol., C.* 42:100338.
5. Watson, W. F., and R. Livingston. 1950. Self-quenching and sensitization of fluorescence of chlorophyll solutions. *J. Chem. Phys.* 18:802–809.
6. Kelly, A. R., and G. Porter. 1970. Model systems for photosynthesis I. Energy transfer and light harvesting mechanisms. *Proc. R. Soc. London, Ser. A.* 315:149–161.
7. Beddard, G. S., S. E. Carlin, and G. Porter. 1976. Concentration quenching of chlorophyll fluorescence in bilayer lipid vesicles and liposomes. *Chem. Phys. Lett.* 43:27–32.
8. MacDonald, R. I. 1990. Characteristics of self-quenching of the fluorescence of lipid-conjugated rhodamine in membranes. *J. Biol. Chem.* 265:13533–13539.
9. Brown, R. S., J. D. Brennan, and U. J. Krull. 1994. Self-quenching of nitrobenzoxadiazole labeled phospholipids in lipid membranes. *J. Chem. Phys.* 100:6019–6027.
10. Dahim, M., N. K. Mizuno, …, H. L. Brockman. 2002. Physical and photophysical characterization of a BODIPY phosphatidylcholine as a membrane probe. *Biophys. J.* 83:1511–1524.
11. Wu, L., C. Huang, T. D. James…, 2020. Förster resonance energy transfer (FRET)-based small-molecule sensors and imaging agents. *Chem. Soc. Rev.* 49:5110–5139.
12. Roy, R., S. Hohng, and T. Ha. 2008. A practical guide to single-molecule FRET. *Nat. Methods.* 5:507–516.
13. Piston, D. W., and G. J. Kremers. 2007. Fluorescent protein FRET: The good, the bad and the ugly. *Trends Biochem. Sci.* 32:407–414.
14. Krüger, T. P. J., and R. van Grondelle. 2017. The role of energy losses in photosynthetic light harvesting. *J. Phys. B Atom. Mol. Opt. Phys.* 50:132001.
15. Ruban, A. V. 2016. Nonphotochemical chlorophyll fluorescence quenching: Mechanism and effectiveness in protecting plants from photodamage. *Plant Physiol.* 170:1903–1916.
16. Ruban, A. V., and S. Wilson. 2021. The mechanism of non-photochemical quenching in plants: Localization and driving forces. *Plant Cell Physiol.* 62:1063–1072.
17. Manna, P., and G. S. Schlau-Cohen. 2022. Photoprotective conformational dynamics of photosynthetic light-harvesting proteins. *Biochim. Biophys. Acta Bioenerg.* 1863:148543.
18. Gray, C., T. Wei, …, C. D. P. Duffy. 2021. Trivial excitation energy transfer to carotenoids is an unlikely mechanism for non-photochemical quenching in LHCII. *Front. Plant Sci.* 12:797373.
19. Azadi-Chegeni, F., M. E. Ward, …, A. Pandit. 2021. Conformational dynamics of light-harvesting complex II in a native membrane environment. *Biophys. J.* 120:270–283.
20. Adams, P. G., C. Vasilev, …, M. P. Johnson. 2018. Correlated fluorescence quenching and topographic mapping of light-harvesting complex II within surface-assembled aggregates and lipid bilayers. *Biochim. Biophys. Acta Bioenerg.* 1859:1075–1085.
21. Son, M., A. Pinnola, …, G. S. Schlau-Cohen. 2020. Observation of dissipative chlorophyll-to-carotenoid energy transfer in light-harvesting complex II in membrane nanodiscs. *Nat. Commun.* 11:1295.
22. Mascoli, V., N. Liguori, …, R. Croce. 2019. Capturing the quenching mechanism of light-harvesting complexes of plants by zooming in on the ensemble. *Chem.* 5:2900–2912.
23. van Amerongen, H., and J. Chmeliov. 2020. Instantaneous switching between different modes of non-photochemical quenching in plants. Consequences for increasing biomass production. *Biochim. Biophys. Acta Bioenerg.* 1861:148119.
24. Tutkus, M., J. Chmeliov, …, L. Valkunas. 2021. Aggregation-related quenching of LHCII fluorescence in liposomes revealed by single-molecule spectroscopy. *J. Photochem. Photobiol., B.* 218:112174.
25. Ruan, M., H. Li, …, Y. Weng. 2023. Cryo-EM structures of LHCII in photo-active and photo-protecting states reveal allosteric regulation of light harvesting and excess energy dissipation. *Nat. Plants.* 9:1547–1557.
26. Hancock, A. M., S. A. Meredith, …, P. G. Adams. 2019. Proteoliposomes as energy transferring nanomaterials: enhancing the spectral range of light-harvesting proteins using lipid-linked chromophores. *Nanoscale.* 11:16284–16292.
27. Hancock, A. M., M. Son, …, P. G. Adams. 2021. Ultrafast energy transfer between lipid-linked chromophores and plant light-harvesting complex II. *Phys. Chem. Chem. Phys.* 23:19511–19524.
28. Hancock, A. M., D. J. K. Swainsbury, …, P. G. Adams. 2022. Enhancing the spectral range of plant and bacterial light-harvesting pigment-protein complexes with various synthetic chromophores incorporated into lipid vesicles. *J. Photochem. Photobiol., B.* 237:112585.
29. Fox, K. F., V. Balevičius, …, C. D. P. Duffy. 2017. The carotenoid pathway: what is important for excitation quenching in plant antenna complexes? *Phys. Chem. Chem. Phys.* 19:22957–22968.
30. Gray, C., L. Kailas, …, C. D. P. Duffy. 2024. Unravelling the fluorescence kinetics of light-harvesting proteins with simulated measurements. *Biochim. Biophys. Acta Bioenerg.* 1865:149004.
31. Knoester, J., and J. E. Van Himbergen. 1987. On the theory of concentration self-quenching by statistical traps. *J. Chem. Phys.* 86:3571–3576.
32. Boulu, L. G., L. K. Patterson, …, J. J. Kozak. 1987. Theoretical investigation of fluorescence concentration quenching in two-dimensional disordered systems. Application to chlorophyll *a* in monolayers of dioleylphosphatidylcholine. *J. Chem. Phys.* 86:503–507.
33. Baumann, J., and M. D. Fayer. 1986. Excitation transfer in disordered two-dimensional and anisotropic three-dimensional systems: Effects of spatial geometry on time-resolved observables. *J. Chem. Phys.* 85:4087–4107.
34. Beddard, G. S., and G. Porter. 1976. Concentration quenching in chlorophyll. *Nature.* 260:366–367.
35. Tamošiūnaitė, J., S. Streckaitė, …, A. Gelzinis. 2023. Concentration quenching of fluorescence in thin films of zinc-phthalocyanine. *Chem. Phys.* 572:111949.
36. Knoester, J., and J. E. Van Himbergen. 1987. Monte Carlo simulations on concentration self-quenching by statistical traps. *J. Chem. Phys.* 86:3577–3582.
37. Shi, W. J., J. Barber, and Y. Zhao. 2013. Role of formation of statistical aggregates in chlorophyll fluorescence concentration quenching. *J. Phys. Chem. B.* 117:3976–3982.
38. Zumofen, G., and A. Blumen. 1982. Energy transfer as a random walk. II. Two-dimensional regular lattices. *J. Chem. Phys.* 76:3713–3731.
39. Bourne-Worster, S., O. Feighan, and F. R. Manby. 2023. Charge transfer as a mechanism for chlorophyll fluorescence concentration quenching. *Proc. Natl. Acad. Sci. USA.* 120:e2210811120.
40. Prlj, A., L. Vannay, and C. Corminboeuf. 2017. Fluorescence quenching in BODIPY dyes: The role of intramolecular interactions and charge transfer. *Helv. Chim. Acta.* 100:e1700093.
41. Marfin, Y. S., E. A. Banakova, …, A. V. Churakov. 2020. Effects of concentration on aggregation of BODIPY-based fluorescent dyes solution. *J. Fluoresc.* 30:1611–1621.
42. Lee, A. G. 1975. Segregation of chlorophyll a incorporated into lipid bilayers. *Biochemistry.* 14:4397–4402.
43. Kelly, A. R., and L. K. Patterson. 1971. Model systems for photosynthesis II. Concentration quenching of chlorophyll *b* fluorescence in solid solutions. *Proc. R. Soc. London, Ser. A.* 324:117–126.
44. Seely, G. R. 1978. The energetics of electron-transfer reactions of chlorophyll and other compounds. *Photochem. Photobiol.* 27:639–654.
45. Meredith, S. A., Y. Kusunoki, …, P. G. Adams. 2023. Self-quenching behavior of a fluorescent probe incorporated within lipid membranes explored using electrophoresis and fluorescence lifetime imaging microscopy. *J. Phys. Chem. B.* 127:1715–1727.

46. Groves, J. T., and S. G. Boxer. 1995. Electric field-induced concentration gradients in planar supported bilayers. *Biophys. J.* 69:1972–1975.

47. Daniel, S., A. J. Diaz, …, P. S. Cremer. 2007. Separation of membrane-bound compounds by solid-supported bilayer electrophoresis. *J. Am. Chem. Soc.* 129:8072–8073.

48. Nabika, H., B. Takimoto, and K. Murakoshi. 2008. Molecular separation in the lipid bilayer medium: Electrophoretic and self-spreading approaches. *Anal. Bioanal. Chem.* 391:2497–2506.

49. van Weerd, J., S. O. Krabbenborg, …, P. Jonkheijm. 2014. On-chip electrophoresis in supported lipid bilayer membranes achieved using low potentials. *J. Am. Chem. Soc.* 136:100–103.

50. Bao, P., M. R. Cheetham, …, S. D. Evans. 2012. On-chip alternating current electrophoresis in supported lipid bilayer membranes. *Anal. Chem.* 84:10702–10707.

51. Roth, J. S., Y. Zhang, …, S. D. Evans. 2015. Optimization of Brownian ratchets for the manipulation of charged components within supported lipid bilayers. *Appl. Phys. Lett.* 106:183703.

52. Hills, R. D., and N. McGlinchey. 2016. Model parameters for simulation of physiological lipids. *J. Comput. Chem.* 37:1112–1118.

53. Förster, T. 1948. Zwischenmolekulare Energiewanderung und Fluoreszenz. *Ann. Phys.* 437:55–75.

54. Chandrasekhar, S. 1943. Stochastic Problems in Physics and Astronomy. *Rev. Mod. Phys.* 15:1–89.

55. Loura, L. M. S. 2012. Simple estimation of Förster Resonance Energy Transfer (FRET) orientation factor distribution in membranes. *Int. J. Mol. Sci.* 13:15252–15270.

56. Sahin, T., M. A. Harris, …, J. S. Lindsey. 2015. Self-assembled light-harvesting system from chromophores in lipid vesicles. *J. Phys. Chem. B.* 119:10231–10243.

57. Lord, S. J., Z. Lu, …, W. E. Moerner. 2007. Photophysical properties of acene DCDHF fluorophores: Long-wavelength single-molecule emitters designed for cellular imaging. *J. Phys. Chem. A.* 111:8934–8941.

58. Zhang, X.-F., and J. Zhu. 2019. BODIPY parent compound: Fluorescence, singlet oxygen formation and properties revealed by DFT calculations. *J. Lumin.* 205:148–157.

59. Meredith, S. A., T. Yoneda, …, P. G. Adams. 2021. Model lipid membranes assembled from natural plant thylakoids into 2D microarray patterns as a platform to assess the organization and photophysics of light-harvesting proteins. *Small.* 17:2006608.

60. Yoneda, T., Y. Tanimoto, …, K. Morigaki. 2020. Photosynthetic model membranes of natural plant thylakoid embedded in a patterned polymeric lipid bilayer. *Langmuir.* 36:5863–5871.

61. Morigaki, K., K. Kiyosue, and T. Taguchi. 2004. Micropatterned composite membranes of polymerized and fluid lipid bilayers. *Langmuir.* 20:7729–7735.

62. Gutschick, V. P. 1978. Concentration quenching in chlorophyll-*a* and relation to functional charge transfer *in vivo*. *J. Bioenerg. Biomembr.* 10:153–170.

63. Descalzo, A. B., P. Ashokkumar, …, K. Rurack. 2020. On the aggregation behaviour and spectroscopic properties of alkylated and annelated boron-dipyrromethene (BODIPY) dyes in aqueous solution. *ChemPhotoChem.* 4:120–131.

64. Selwyn, J. E., and J. I. Steinfeld. 1972. Aggregation of equilibriums of xanthene dyes. *J. Phys. Chem.* 76:762–774.

65. Knox, R. S. 1994. Spectral effects of exciton splitting in "statistical pairs". *J. Phys. Chem.* 98:7270–7273.

66. Ottiger, P., H. Köppel, and S. Leutwyler. 2015. Excitonic splittings in molecular dimers: Why static ab initio calculations cannot match them. *Chem. Sci.* 6:6059–6068.

67. Zhegalova, N. G., S. He, …, M. Y. Berezin. 2014. Minimization of self-quenching fluorescence on dyes conjugated to biomolecules with multiple labeling sites via asymmetrically charged NIR fluorophores. *Contrast Media Mol. Imaging.* 9:355–362.

68. Wan, C., T. Xia, …, A. H. Zewail. 2005. Ultrafast unequilibrated charge transfer: A new channel in the quenching of fluorescent biological probes. *Chem. Phys. Lett.* 412:158–163.

69. Niedzwiedzki, D. M., C. N. Hunter, and R. E. Blankenship. 2016. Evaluating the nature of so-called S*-state feature in transient absorption of carotenoids in light-harvesting complex 2 (LH2) from purple photosynthetic bacteria. *J. Phys. Chem. B.* 120:11123–11131.

70. Wahadoszamen, M., R. Berera, …, R. van Grondelle. 2012. Identification of two emitting sites in the dissipative state of the major light harvesting antenna. *Phys. Chem. Chem. Phys.* 14:759–766.

71. Meredith, S. A., Y. Kusunoki, P. G. Adams…, 2024. Dataset for the Study of Evidence for a Transfer-To-Trap Mechanism of Fluorophore Concentration Quenching in Lipid Bilayers. University of Leeds. https://doi.org/10.5518/1480.

72. Krider, E. P. 2013. The Nearest Neighbor Probability Distribution. University of Arizona, ATMO489, Atmospheric Electricity. http://www.atmo.arizona.edu/students/courselinks/spring13/atmo589/ATMO489_online/lecture_19/lect19_nearest_neighbor_dist.html.

73. Jordan, D., and P. Smith. 2008. Mathematical Techniques: An Introduction for the Engineering, Physical, and Mathematical Sciences. Oxford University Press, Oxford, UK.

74. Martin, B. R., and G. Shaw. 2015. Mathematics for Physicists. Wiley, Chichester, UK.

75. Johansson, L. B. Å., and G. Lindblom. 1986. Application of time-resolved fluorescence in the study of lipid aggregates II. Motions and order of pyrene probes in an aligned lyotropic nematic phase. *Liq. Cryst.* 1:53–62.

76. Wistus, E., E. Mukhtar, and L. B. Å. Johansson. 1996. A conspicuous orientation and localization of pyrene in Langmuir lipid monolayers. *Langmuir.* 12:3371–3373.

Biophysical Journal, Volume *123*

Supplemental information

Evidence for a transfer-to-trap mechanism of fluorophore concentration quenching in lipid bilayers

Sophie A. Meredith, Yuka Kusunoki, Stephen D. Evans, Kenichi Morigaki, Simon D. Connell, and Peter G. Adams

Supplemental Information

for:

Evidence for a transfer-to-trap mechanism of fluorophore concentration quenching in lipid bilayers

Sophie A. Meredith,[1,2] Yuka Kusunoki,[3] Stephen D. Evans,[1]
Kenichi Morigaki,[3] Simon D. Connell,[1,2] Peter G. Adams[1,2,*]

[1] School of Physics and Astronomy, University of Leeds, Leeds, LS2 9JT, U. K.

[2] Astbury Centre for Structural Molecular Biology, University of Leeds, Leeds, LS2 9JT, U. K.

[3] Graduate School of Agricultural Science and Biosignal Research Center, Kobe University Rokkodaicho 1-1, Nada, Kobe, 657-8501, Japan

[*]correspondence: p.g.adams@leeds.ac.uk

SI 1. Finding the average distance between molecules from a nearest neighbour distribution

In the main text, the average distance between fluorophores is estimated from the average area occupied per molecule, as follows:

$$\text{Effective area per molecule} = \frac{1}{C_N} = \pi r^2 \qquad \text{Main text Eq. 2}$$

where C_N is the concentration of fluorophores in molecules per nm^2 and r is the average distance between molecules. This can be rearranged to:

$$r = \pi^{-\frac{1}{2}} C_N^{-\frac{1}{2}} \qquad \text{Main text Eq. 3}$$

which is approximately:

$$\boldsymbol{r = 0.564\, C_N^{-1/2}} \qquad \textbf{Eq. S1}$$

This expression was considered in several other publications as a perfectly valid approximation of the average distance between molecules in a random distribution, including in the theoretical framework of Porter et al. (1, 2) that our theory section is based upon.

Alternative expressions for the average distance between molecules may consider a statistical distribution of distances between nearest neighbours, for example, Chandrasekhar (3). Below, we provide a derivation adapted and expanded from the 3-D case given by Chandrasekhar (3), with detail on random 2-D distributions by Krider (4) and standard textbooks (5, 6). The situation under consideration is shown in **Fig. S1**.

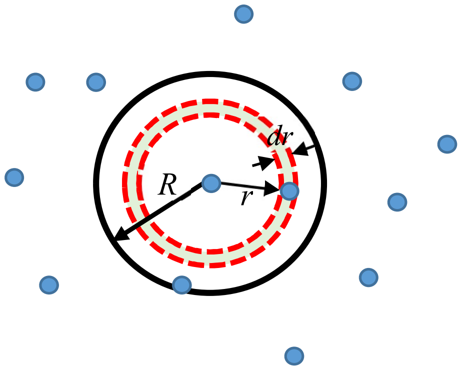

Figure S1: *Schematic of a random 2-D distribution of particles. For each particle, we can consider the probability of a second particle occurring at a range of distances (r) from it.*

The probability that a random particle will be found between distances *r* and (*r* + *dr*) in a circle of radius *R,* with all positions equally likely (so *f(r)* is a constant, *k*) is:

$$f(r) 2\pi r\, dr \qquad \text{Eq. S2}$$

where *f(r)* = *k.* Normalise the distribution function to ensure that integrating over all possible values of r is 1: $\int_{-\infty}^{\infty} f(x)dx = 1$

$$2\pi k \int_0^R r\, dr = \frac{2\pi k R^2}{2} = 1$$

$$k = \frac{1}{\pi R^2} \qquad \text{Eq. S3}$$

Hence the probability that a single particle will be found between *r* and *r* + *dr* is given by

$$f(r)dr = \frac{2\pi r\, dr}{\pi R^2} \qquad \text{Eq. S4}$$

For N particles (given each is independent/random), the chance that a particle is between r and $r + dr$ is N times above function.

$$f(r) = \frac{N\ 2\pi r\ dr}{\pi R^2} = 2\pi rn\ dr \qquad \text{Eq. S5}$$

Where $n = N/\pi R^2$, the average number of particles per unit area (i.e., particle number density). Let $w(r)dr$ denote the probability that a particle is found between r and dr , but at the same time no other particles exist in the space inside of circle r = the definition of a nearest neighbour. This will equal the probability of being between r and $r + dr$ times the probability of no particles being found inside of r.

$$w(r)dr = 2\pi rn\ dr \left[1 - \int_0^r w(r)dr\right] \qquad \text{Eq. S6}$$

The integral of $w(r)$ from 0 to r finds the probability that a particle is inside r, so to find the probability another particle does not exist inside of r, we subtract from 1. The $2\pi r.dr.N$ term finds the probability of the nearest neighbour particle existing in the area between r and $r + dr$.

Now need to solve for $w(r)$, but $w(r)$ is also in the integral. Step 1 is to differentiate Eq. S6 with respect to r.

$$\begin{aligned}\frac{d\,w(r)}{dr} &= 2\pi rn\ \frac{d}{dr}\left(1 - \int_0^r w(r')dr'\right) + 2\pi n\left(1 - \int_0^r w(r')dr'\right)\\ &= 2\pi rn\ \left(-\frac{d}{dr}\int_0^r w(r')dr'\right) + 2\pi n\left(1 - \int_0^r w(r')dr'\right)\end{aligned} \qquad \text{Eq. S7}$$

but r appears in the limits of the integral term we need to differentiate. To solve we must apply Leibnitz's rule:

$$-\frac{d}{dr}\int_{f1(r)}^{f2(r)} g(t)dt = -\left[g\big(f_2(r)\big)\frac{d}{dr}\big(f_2(r)\big) - g\big(f_1(r)\big)\frac{d}{dr}\big(f_1(r)\big)\right] \qquad \text{Eq. S8}$$

$$-\frac{d}{dr}\int_0^r g(t)dt = -[w(r)(1) - w(0)(0)] = -w(r) \qquad \text{Eq. S9}$$

Substitute Eq. S9 into Eq. S7

$$\frac{d\,w(r)}{dr} = 2\pi rn\ (-w(r)) + 2\pi n\left(1 - \int_0^r w(r')dr'\right) \qquad \text{Eq. S10}$$

Then, if we rearrange Eq. S6 to give

$$\frac{w(r)\cancel{dr}}{2\pi r\ \cancel{dr}\ n} = \left[1 - \int_0^r w(r)dr\right] \qquad \text{Eq. S11}$$

We substitute this resulting Eq. S11 back into Eq. S10 to give:

$$\frac{d\,w(r)}{dr} = 2\pi rn\ (-w(r)) + \cancel{2\pi n}\frac{w(r)}{\cancel{2\pi rn}} \qquad \text{Eq. S12}$$

$$= -2\pi rn\,w(r) + \frac{w(r)}{r} \qquad \text{Eq. S13}$$

$$\frac{d\,w(r)}{dr} = w(r)\left(\frac{1}{r} - 2\pi rn\right) \qquad \text{Eq. S14}$$

$$\frac{d\,w(r)}{w(r)} = \left(\frac{1}{r} - 2\pi rn\right) dr \qquad \text{Eq. S15}$$

$$\int \frac{d\,w(r)}{w(r)} = \int \left(\frac{1}{r} - 2\pi rn\right) dr \qquad \text{Eq. S16}$$

Which is now solvable.

$$\int \frac{d\,w(r)}{w(r)} = \int \left(\frac{1}{r} - 2\pi rn\right) dr \qquad \text{Eq. S17}$$

$$\ln w(r) = \ln(r) - \pi r^2 n + c \qquad \text{Eq. S18}$$

$$\ln\left[\frac{w(r)}{r}\right] = -\pi r^2 n + c$$

Eq. S19

$$\frac{w(r)}{r} = ke^{-\pi r^2 n}$$

Eq. S20

$$w(r) = kre^{-\pi r^2 n}$$

Remove unknown constant k by normalising the equation.

$$\int_0^\infty w(r)\,dr = 1 = k\int_0^\infty re^{-\pi r^2 n}\,dr \qquad \text{Eq. S21}$$

$$1 = \frac{-k}{2\pi n} e^{-\pi r^2 n} \Big|_0^\infty \qquad \text{Eq. S22}$$

$$k = 2\pi n \qquad \text{Eq. S23}$$

Substitute the constant from Eq. 23 into Eq. S20 to give the nearest neighbour distribution function, which is a version of the Poisson distribution.

$$\boldsymbol{w(r) = 2\pi rn\, e^{-\pi r^2 n}} \qquad \textbf{Eq. S24}$$

The exact formula for the average distance D between particles can now be found.

$$D = \int_0^\infty rw(r)\,dr \qquad \text{Eq. S25}$$

$$D = \int_0^\infty 2\pi r^2 n\, e^{-\pi r^2 n} \qquad \text{Eq. S26}$$

We use integration by substitution. In general

$$\int_a^b f(x)\,dx = \int_{z(a)}^{z(b)} f(x[z]) \frac{dx[z]}{dz} dz$$

(note *dx* would be *dr* and we substitute in $z = \pi r^2 n$ into (25):

$$D = \int_0^\infty 2\pi r^2 n\, e^{-z} \frac{1}{2\pi rn}\,dz \qquad \text{Eq. S27}$$

n.b. differentiate $dz/dr = 2\pi rn$, (where $dx = dr$ in this case), then $dx/dz = 1/2\pi rn$

$$D = \int_0^\infty \cancel{2\pi} r^{\cancel{2}} \cancel{n}\, e^{-z} \frac{1}{\cancel{2\pi rn}}\,dz \qquad \text{Eq. S28}$$

$$D = \int_0^{\infty} r\, e^{-z}\, dz \qquad \text{Eq. S29}$$

And if $z = \pi r^2 n$ then $r = \sqrt{\frac{z}{\pi n}}$, hence

$$D = \int_0^{\infty} \frac{z^{\frac{1}{2}}}{(\pi n)^{\frac{1}{2}}} e^{-z}\, dz \qquad \text{Eq. S30}$$

$$D = \frac{1}{(\pi n)^{\frac{1}{2}}} \int_0^{\infty} e^{-z} z^{\frac{1}{2}}\, dz \qquad \text{Eq. S31}$$

The integral term can be solved using the gamma function, i.e.

$$\Gamma(n) = \int_0^{\infty} e^{-x} x^{n-1}\, dx = (n-1)!$$

To translate our function into the correct form, if $n - 1 = \frac{1}{2}$ then $n = \frac{3}{2}$

$$D = \frac{1}{\sqrt{\pi n}} \Gamma\left(\frac{3}{2}\right) \qquad \text{Eq. S32}$$

Using an online calculator to find the gamma function result $\Gamma\left(\frac{3}{2}\right) = 0.8862269$, which is equal to $\frac{\sqrt{\pi}}{2}$

$$D = \frac{1}{\sqrt{\pi}} \frac{\sqrt{\pi}}{2}\, n^{-\frac{1}{2}} \qquad \text{Eq. S33}$$

$$D = 0.5 n^{-\frac{1}{2}} \qquad \text{Eq. S34}$$

Or, in the generalized notation used in the main text, where $D = r$ and $n = C_N$:

$$\boldsymbol{r = 0.5 C_N^{-\frac{1}{2}}} \qquad \text{Eq. S35}$$

We can now compare the two different expressions that estimate the average distance between molecules in the random 2-D distribution. Overall, there is a difference of approximately 11% between the value for *r* calculated by Eq. S35 ($r = 0.5\ C_N{}^{-1/2}$) compared to Eq. 3/ Eq. S1 ($r = 0.564\ C_N{}^{-1/2}$). This is a relatively small difference, given the other uncertainties in this work, and is not significant enough to affect any of our conclusions. Therefore, we prefer to use Eq. 3 in the main text, to be consistent with previous publications on transfer-to-trap theory.

We can use the expression for the statistical distribution to generate graphs (see **Fig. S2**, below). Here, the peaks of the distributions are at slightly (~10%) lower distance values than the average distance as calculated by Eq. S35. This is due to the right-skewed Poisson distribution which describes the random arrangement of fluorophores. In the current study, our theoretical treatment of quenching considers the *average* distance between fluorophores rather than directly evaluating distributions of *r*. This is reasonable because the later equations based on *r*, such as "fraction of traps" generated for a given concentration, consider the probabilities of processes occurring based on a distribution, rather than a definite result. It may be interesting to consider distributions of distances in more detail in future work.

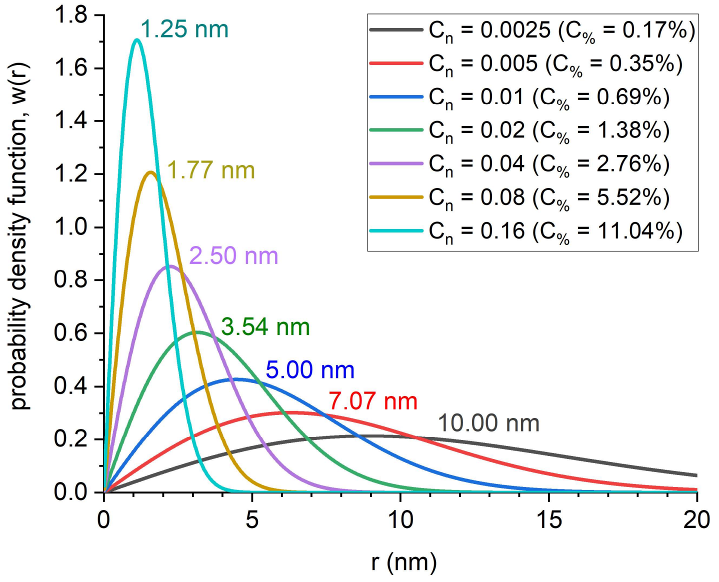

Figure S2: *Statistical distribution of distances between nearest-neighbour molecules, as calculated by Eq. S24. The probability density function w(r) is plotted for a range of concentrations of fluorophore (C_n). The numbers shown the next to each peak correspond to the value for r calculated by Eq. S35.*

SI 2. Confirming the membrane quality: structural contiguity and lateral lipid diffusion

The FLIM instrumentation was set up as described in the **Experimental Methods** section (main text), changing the excitation laser and emission filters used so that they were appropriate for the fluorophore. For TR excitation at 561 nm and collection between 590-650 nm was used, whereas, for NBD and BODIPY excitation at 485 nm and collection 500-540 nm was used. FLIM images of lipid membranes containing 0.5% (w/w) NBD, TR, and BODIPY are shown in **Figure S3A**. In each case, fluorescence is restricted to well-defined square patterned membranes, with minimal signal located on the surrounding template. The fluorescence within each membrane is largely homogeneous across the corral (40-50 counts/pix for all fluorophores), with a few visible bright spots that may represent non-ruptured vesicles that are loosely adsorbed onto the membrane. Overall, these membranes were highly reproducible, with minimal variation in intensity and quality across multiple preparations.

To confirm that the liposomes had ruptured to form well-connected membranes containing mobile fluorophores (and not merely adsorbed into the template without rupturing), fluorescence recovery after photobleaching (FRAP) experiments were performed to monitor the diffusion of lipids over time. For each sample, a circular area (with a bleached radius, R_{bleach}, ranging from 20-30 μm) of membrane was deliberately photobleached using intense white light. Immediately after photobleaching, a FLIM timelapse of images (**Figure S3B**) was obtained to monitor the diffusion of "non-bleached" fluorophores into the bleached area. The intensity of the fluorescence recovery in the bleached spot was monitored for each timepoint (each time point is the accumulation of photons in a 16 s period) to plot a fluorescence recovery curve (**Figure S3C**). A mono-exponential fit, $F = F_0(1 - e^{-kt})$, was used to obtain the rate constant k and then the "doubling time", $\tau = ln(2)/k$, for each sample. From this, the diffusion constant, $D = 0.22 \times R_{bleach}^2/\tau$, was calculated. To calculate the mobile fraction, images of the corral before photobleaching and after photobleaching were analysed. Briefly, the intensity of the "bleached" region was compared to the intensity of a "non-bleached" region throughout the FRAP experiment. For a mobile fraction of 100% the two regions would have an equal intensity after the system has been allowed to reach an equilibrium. A summary of FRAP experiments on patterned bilayers is shown in **Table S1.** For each fluorophore, the diffusion of the lipids was compared in both patterned bilayers (lipid bilayers ruptured into a 100 μm square template) and in "infinite" bilayers (lipid bilayers ruptured onto non-patterned glass) to ensure that the Diyne-PC template did not adversely affect the lipid mobility. The calculated diffusion constant was similar for all fluorophores in both patterned and non-patterned bilayers (ranging from 1.75 to 2.27 $\mu m^2/s$) and the mobile fraction was consistently high across all samples (>94% for all fluorophores). Overall our results are consistent with the values for the diffusion constant and mobile fractions of similar lipid-tagged fluorophores reported in other studies (7, 8), and a strong indication that any interactions that the lipid-tagged fluorophores may have with either the glass substrate or the Diyne-PC template do not hinder the lateral diffusion of lipids or the electrophoretic effect. In addition, the mobility, $\mu_{FRAP} = D/k_BT$, was calculated for each fluorophore (ranging between 4.2 to 5.5 $ms^{-1}N^{-1}$). The mobility is a measure of how a particle responds to an applied force, and will be used to quantify the electrophoretic effect investigated, see **SI 4**.

The accidental photobleaching of fluorophores by the excitation laser could have the effect of altering the concentration of "active fluorophores", which would be undesirable and lead to misinterpretation of fluorescence microscopy data. Photobleaching will increase with laser power and acquisition time. Therefore, the photobleaching of the fluorescent probes was quantified and minimized, as described in reference (9). Similarly, singlet-singlet excited state annihilation effects can distort fluorescence measurements by reducing the values measured for fluorescence lifetime. The probability of annihilation effects increases with laser power or, more accurately, the energy per pulse per unit area (illumination area on the sample surface). Therefore, a laser power dependence measurement series was made to quantify annihilation effects for these fluorescent probes, as shown in reference (9). As found in that publication, the optimal laser fluence for this instrument and these type of samples was found to be ~0.012 mJ cm^{-2} with a laser spot diameter of ~800 nm (10 MHz repetition rate, pulse widths of ~70-100 ps for the 485 nm laser and 561 nm laser). With these laser settings, there were no detectable annihilation effects and photobleaching over the course of a standard FLIM image acquisition of 25 frames (80 s of exposure) was below 2%, for all fluorescent probes used.

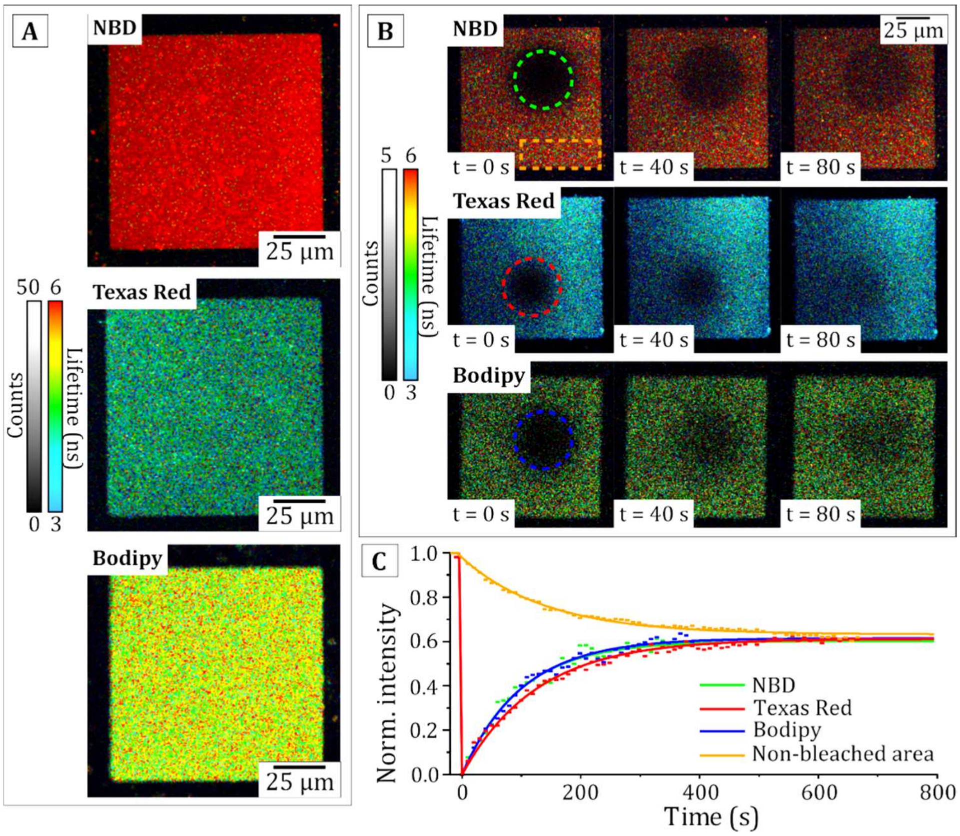

Figure S3: *FLIM images of TR-, NBD- and BODIPY-containing lipid bilayers and Fluorescence Recovery After Photobleaching (FRAP) experiments confirming that the fluorophores are mobile and a suitable target for electrophoresis.* ***(A)*** *Example images of DOPC lipid bilayers containing 0.5% (mol/mol) of either NBD (top), TR (middle) or BODIPY (bottom) formed as 100 µm wide corrals within the DiynePC polymer templates. These are all set to the same fluorescence lifetime scale of 3-6 ns to allow a visual comparison of the relative differences in lifetime.* ***(B)*** *FRAP experiments for the patterned lipid bilayers, showing the mobility of each type of fluorophore.* ***(C)*** *FRAP recovery curves showing the fluorescence intensity in the dashed bleached regions in (b), normalised to the intensity of the bilayer prior to photobleaching (colours as labelled on legend). The orange curve shows the fluorescence intensity decrease in the orange, box region in (b), showing that the intensity in the patterned membrane decreases due to there being a finite number of non-bleached fluorophores available to diffuse into the bleached region. The FRAP data on TR is reproduced from Meredith et al. 2023 (9) (the other data is new).*

	**TR**		**NBD**		**BODIPY**	
	Patterned bilayer	Infinite bilayer	Patterned bilayer	Infinite bilayer	Patterned bilayer	Infinite bilayer
D ($\mu m^2 s^{-1}$)	2.27 ± 0.59	1.95 ± 0.20	2.13 ± 0.18	1.99 ± 0.20	2.09 ± 0.39	1.75 ± 0.17
μ_{FRAP} ($ms^{-1}N^{-1}$)	5.49 ± 0.14	4.71 ± 0.48	5.15 ± 0.43	4.81 ± 0.48	5.05 ± 0.94	4.23 ± 0.41
Mobile %	95.5 ± 2.5	96.6 ± 3.6	94.7 ± 2.3	97.0 ± 5.2	94.0 ± 1.8	95.3 ± 7.2

Table S1: *Summary of FRAP experiments comparing patterned lipid bilayers (SLBs in a 100 µm square corrals) versus non-pattered lipid bilayers (SLBs on piranha-cleaned glass coverslips). Overall, the similarity of the results between different substrates indicates that the prescence of the polymerized lipid (Diyne-PC) template has no significant effect on the mobility of lipids close to the centre of the membrane.*

SI 3. Consideration of the relative orientation of fluorophores

The orientation of fluorophores should be carefully considered because this can affect the efficiency of excitation energy transfer and could affect trap formation. The following arguments led us to the conclusion that there was unlikely to be any alignment of fluorophores and that, even if there was, this would not make a significant difference to our findings:

1. Many of the previous studies that observed polarization of fluorophores were assessing fluorophores such as pyrene that embed within the hydrophobic portion of the lipid bilayer (10, 11). These embedded fluorophores experience strong interactions with the fatty acyl chains of lipid (which do have significant alignment), whereas, this is not the case for fluorophores that are conjugated to the headgroup of a lipid.
2. The TR/ NDB/ BODIPY fluorophores used in our work are not likely to have long-lived orientational alignment because they are tethered to the headgroup of (DHPE) lipids and are therefore likely to reside outside of the lipid membrane, where they are free to reorient, unimpeded by any alignment of lipid fatty acyl chains. There is a linker of three carbons in length between the lipid's phosphate group and the fluorophore (see **Figure 2D** in the main text) and there will be free rotation along the length of the sigma-bonds in this linker region and additional twisting in all directions due to the multiple segments. This freedom means that the lipid-fluorophore conjugate can experience many different conformations. Thus, the transition dipole of the fluorophore is able to reorient in so many different ways that it approximates to being isotropic for the purposes of excitation energy transfer and Förster theory (i.e., $\kappa^2 \sim 2/3$).
3. Even if we discard the arguments given in [1] and [2] and assume that our fluorophores did have a preference for alignment with the lipid, this would only have a limited effect on FRET efficiency, as explained below. The orientation factor κ^2, can vary between $\kappa^2 = 0$ for perfectly perpendicular transition dipoles and $\kappa^2 = 1$ for perfectly co-planar transition dipoles (κ^2 can increase higher if fluorophores are aligned end-to-end but this is not possible for our system). In the main text, we assume a value of $\kappa^2 = 2/3$ (random orientation). If κ^2 had a preference for alignment it could lie between 0.67 and 1.00, for example, a value 0.83 would be intermediate between these values. This is increase of 16% for κ^2 would lead to an increase in Förster radius of only 7%, as $(R_0)^6 \propto (\kappa^2)$ (main text Eq. 12). This equates to a nonlinear increase in FRET efficiency by a maximum of 10% (close to the Förster radius the efficiency increases from 40-50% to 50-60%).
4. The theoretical model for the transfer-to-trap mechanism for fluorophore quenching used in the main text (Eq. 5 – Eq. 23) shows that differences in FRET efficiency have very minimal effect on the overall efficiency of trapping. This is because the trapping efficiency is dominated by the formation of trap sites, as described in the text and shown in **Figure 1E** (in the main text) and **Figure S7** (in the supporting info). Therefore, even the hypothetical increase in FRET efficiency occurring in the "worst case" scenario of point [3] would have limited effect on overall trapping efficiency.
5. The only remaining "unknown" would be the possible effect of fluorophore alignment on trap formation (statistical dimers). Again, if we discard the arguments given in [1] and [2] and assume that our fluorophores have some alignment with the lipid, which we think is unlikely, then this could increase the likelihood of trap formation. This would not change the trends that we observe or our conclusions but it would simply scale the critical radius (R_C) values calculated by some modest amount.

Future work could investigate polarization effects to attempt to prove or disprove these assumptions.

SI 4. Full calculation of electrophoretic drift velocity for all TR, NBD and BODIPY

A series of time-lapse measurements are shown for corrals of SLBs containing TR/DOPC (**Figure S4A-C**). The drift velocity for TR was found by fitting a straight line (*blue line,* **Figure S4D**) to the moving edge displacement plot and found to be 0.28 ± 0.01 μm/s (fitted value ± uncertainty). The analysis of TR was recently published in another paper from our group (9). The analysis process was repeated for NBD and BODIPY and the fluorophore mobility was calculated (**Figure S4E**). A summary of the calculated kinetic parameters for all three fluorophores is shown in **Table S2**. To determine the contribution of electroosmotic drag to the mobility of fluorophores, the electrophoretic mobility, $\mu_{EP} = V_d / qeE$, was calculated and compared to the FRAP mobility, μ_{FRAP}, for each NBD, TR and BODIPY (from **Figure S3** and **Table S1**). The ratio of the two mobilities, $\alpha = \mu_{EP}/\mu_{FRAP}$, was calculated for each fluorophore to describe the reduction in the mobility due to electroosmotic drag. The reduction factor was found to be 0.83 ± 0.10, 0.70 ± 0.04 and 0.74 ± 0.02 for NBD, TR and BODIPY respectively. These values are similar to the values for α found in other studies of electrophoresis of lipid tagged fluorophores (8), and show that all three fluorophores are affected by electroosmotic drag to some degree. The different reduction factor for each fluorophore is likely due to the amount that each fluorophore protrudes into the aqueous buffer, for example, a large, bulky molecule will experience more friction and drag from the ions close to the membrane. Characterising the kinetics of electrophoresis in this manner shows that it is possible to control the re-organisation of fluorophores, with similar success for each fluorophore.

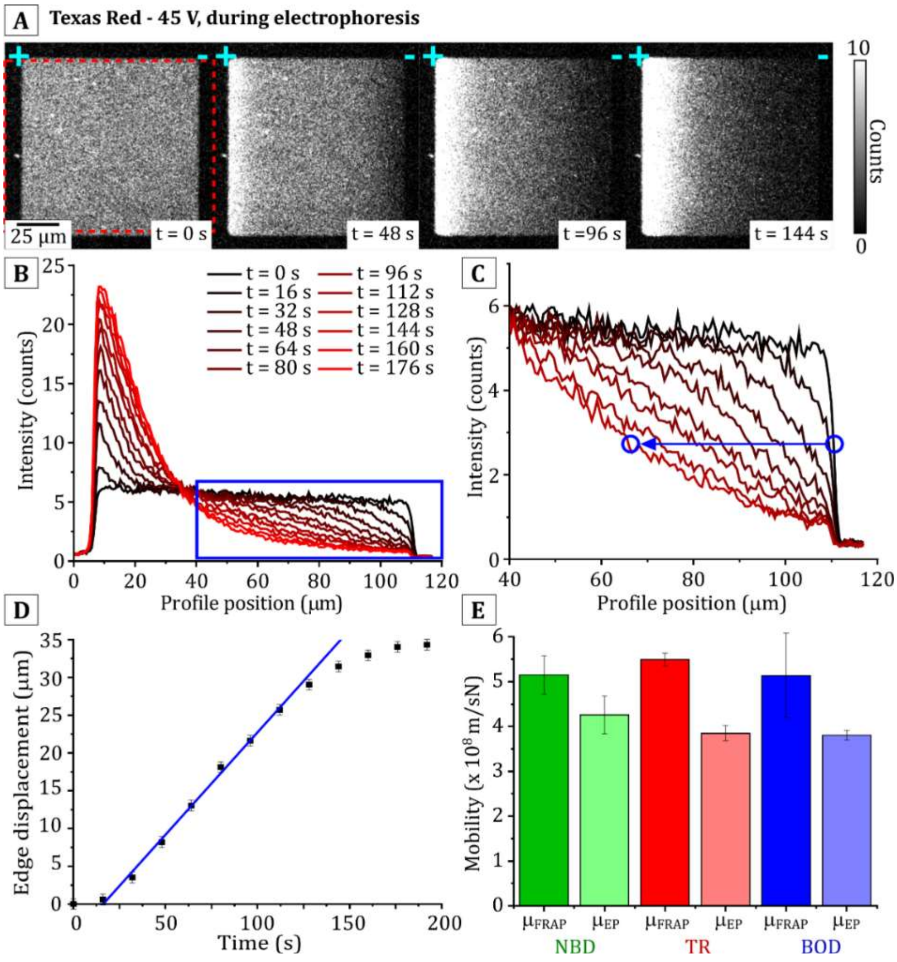

Figure S4: *Timelapse analysis of the drift velocity of TR, NBD and BODIPY.* ***(A)*** *Timelapse series of FLIM images (intensity-only) of a bilayer containing 0.5% (w/w) TR-DHPE after the application of an electric field (45 V/cm).* ***(B)*** *Average intensity profiles measured in the red, dashed box region in panel (a). Black-to-red lines represent increasing timepoints separated by 16 s intervals.*

Figure S4 (caption continued): (C) *A higher magnification portion of panel (b), focusing specifically on the "moving edge" at the right side of the patterned bilayer. The edge midpoint (position of the half-maximum intensity) is measured (blue circle) for each timepoint.* ***(D)*** *Graph showing the displacement of the moving edge of fluorescence with increasing timepoints. The drift velocity, V_{drift}, is obtained from a linear fit (blue line).* ***(E)*** *Graph showing the fluorophore mobility as measured by FRAP, μ_{FRAP}, and as measured through by electrophoresis, μ_{EP} for NBD (green), TR (red), and BODIPY (blue). Panels A-D are reproduced from Meredith et al. 2023 (9).*

Fluorophore	μ_{FRAP} ($\times 10^8$ m/sN)	v_d (μm/s)	***q*** (e)	***E*** (V/μm)	μ_{EP} ($\times 10^8$ m/sN)	***α***
TR	5.49 ± 0.14	0.31 ± 0.03	-1	0.0045	3.85 ± 0.17	0.70 ± 0.04
NBD	5.15 ± 0.43	0.28 ± 0.01	-1	0.0045	4.26 ± 0.42	0.83 ± 0.10
BODIPY	5.14 ± 0.94	0.27 ± 0.08	-1	0.0045	3.81 ± 0.11	0.74 ± 0.02

Table S2: *Summary of the calculation of kinetic parameters for diffusion of TR, NBD and BODIPY.*
μ_{FRAP} is the Einstein mobility of each fluorophore as in Table S1;
v_d is the drift velocity of each fluorophore as estimated in Figure S4D;
q is the charge per molecule at neutral pH in units of e (elementary charge, 1.602×10^{-19} C);
E is the electric field strength applied (measured during the experiment with a voltmeter);
μ_{EP} is the electrophoretic mobility of each fluorophore.
α is the ratio of electrophoretic and Einstein mobilities, $\alpha = \mu_{EP}/\mu_{FRAP}$.
For fluorophores measured in a previous study of TR electrophoresis (8), the electrophoretic mobility was found to be 40% lower than the Einstein mobility due to the effect of electroosmotic drag (i.e., α ≈ 0.6). Here we found that μ_{EP} was 20-30% lower (i.e., α ≈ 0.7-0.8).

SI 5. Conversion of raw fluorescence intensity data to molecular concentration by using a standard curve and correcting for the effects of quenching

It is important for our analysis of quenching to be able to quantify the concentration of the molecules of interest. In a recent study (9), we showed that it is possible to convert experimentally-measured values for fluorescence intensity into the molecular concentration of the fluorescent molecule (Texas Red). Similar analysis was performed on NBD and BODIPY, in addition to TR, as described below.

Supported lipid bilayers (SLBs) were formed on hydrophilic glass, containing different fluorophore concentrations in a range from 0.25-1.5% weight of fluorophore relative to weight of total lipids, of either TR, NBD or BODIPY. We later realized that units of "mole-to-mole" for concentrations were required for the theoretical modelling, therefore, each weight-to-weight concentration was converted to a mole-to-mole percentage before performing the analysis. These SLBs (without any template pattern) acted as simple control samples, due to their high reproducibility, high throughput, and the ability to accurately prepare a defined concentration of fluorophores. FLIM analysis was performed on the SLBs using the same image acquisition parameters as described for electrophoresis measurements on membrane corrals so that the fluorescence intensities and lifetimes were perfectly comparable. FLIM images for these samples are displayed in **Figure S5A-C** and show that the fluorescent lipids were homogeneously distributed across the SLB, as expected. The average fluorescence intensity and average fitted lifetime was calculated for each image. The raw data for the observed fluorescence intensity (datapoints labelled *F* in **Figure S5D-F**) was found to increase non-linearly with fluorophore concentration for TR, NBD and BODIPY, due to the increasing effect of quenching that occurred at higher concentrations. Concurrent with this, the fluorescence lifetime was found to gradually decrease as fluorophore concentration increased (datapoints labelled τ in **Figure S5D-F**)

In the absence of quenching, the fluorescence intensity would increase linearly with concentration because, logically, with 2× the density of molecules there will be 2× the number of excited states generated for any given excitation laser pulse and, thus, 2× the number of photons of fluorescence expected. This would be a simple relationship from which to estimate the fluorophore concentration, however, this is not observed in the raw data because the fluorescence intensity is somewhat reduced as a result of quenching. As defined in the main text Eq. 16, Quenching Efficiency, $QE = 1 - \tau/\tau_0$, so the reduction in fluorescence lifetime is a measure of quenching. The fluorescence lifetime (τ) measured in a FLIM image was used to calculate the relative change in lifetime, τ/τ_0, where τ_0 is the lifetime in the absence of any quenching (here, τ_0 was defined as the lifetime measured for the fluorophore within an SLB at 0.25%). This ratio τ/τ_0 was then used to calculate the "non-quenched" fluorescence intensity (F_0) from the raw fluorescence intensity (F), according to the expression derived in the main text:

$$F_0 = F \cdot e^{\left[2\ln\left(\frac{\tau_0}{\tau}\right)\right]} \qquad \text{Main text Eq. 23}$$

The result of this calculation is shown in **Table S3** and plotted for each fluorophore in **Figure S5D-F** (datapoints labelled *F0*). As expected from our logic that fluorescence must be proportional to concentration in an ideal system, the "corrected fluorescence intensity" F_0 does indeed appear to have a linear increase with the concentration and was successfully fit to a straight line, $F_0 = mC + Y_0$, where m is the fitted gradient and Y_0 is the y-intercept ($R^2 > 0.99$ for all three datasets). The finding that there is a good linear fit is excellent evidence that the method to estimate the non-quenched fluorescence is accurate. The relationships between the corrected fluorescence intensity and fluorophore concentration were found to be $F_0 = 168.3 \times C_{TR} + 4.8$, $F_0 = 73.0 \times C_{NBD} + 9.7$, and $F_0 = 168.8 \times C_{BODIPY} + 15.3$ for TR, NBD and BODIPY respectively. The fact that Y_0 is not equal to zero for all three fluorophores is likely to be due to a low amount of fluorescence background in the corral region. Using these relationships, the fluorescence intensity and the fluorescence lifetime in FLIM images were used to calculate the fluorophore concentration at each location in corrals before or after electrophoresis.

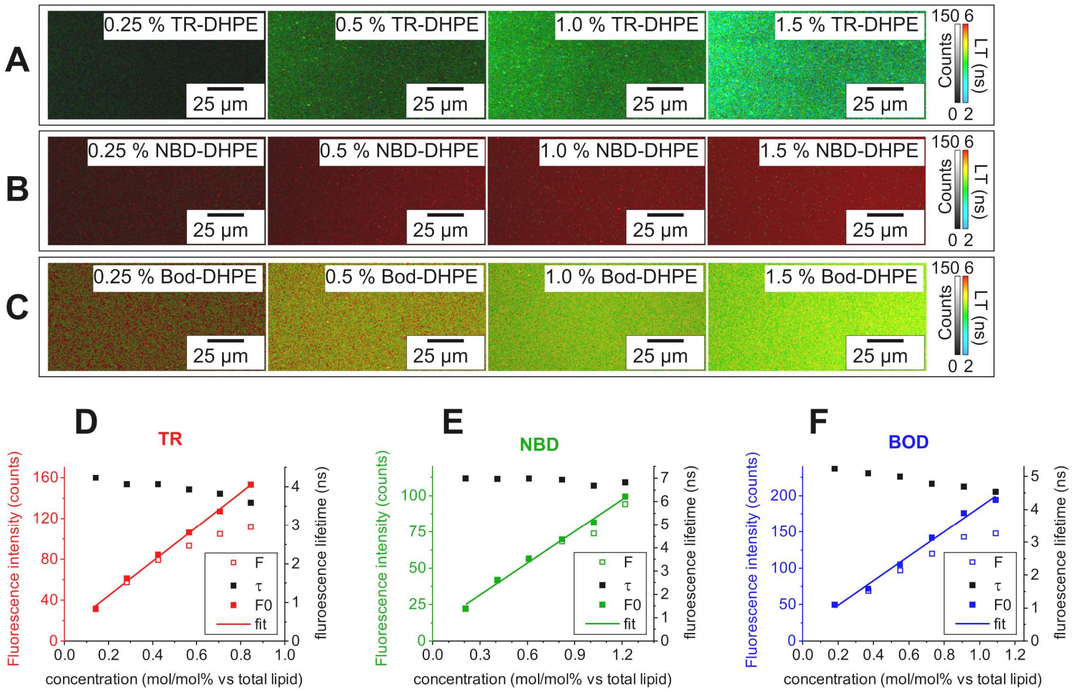

Figure S5: *FLIM images of lipid bilayer standard samples containing fluorophores at defined concentrations used to generate standard curves from which to estimate fluorophore concentrations.* ***(A)*** *Example FLIM images of a series of SLBs containing increasing concentrations of TR-DHPE (concentrations in weight/weight, as labelled). FLIM images were obtained using the same settings as for in-membrane electrophoresis experiments.* ***(B)*** *Example FLIM images of a series of lipid bilayers containing increasing concentrations of NBD-DHPE.* ***(C)*** *Example FLIM images of a series of lipid bilayers containing increasing concentrations of BODIPY-DHPE.* ***(D)-(F)*** *The raw fluorescence intensity (F) observed within the sample (counts/pix) plotted against the known fluorophore concentration of the SLB. Corrected "non-quenched" fluorescence intensity (F_0) was calculated from F using main text Eq. 23. τ was plotted as measured from the FLIM data. All numerical data for F, F_0 and τ is in* ***Table S3****.*

Sample	Conc. (% wt/wt)	Conc. (%mol/mol)	*F* (counts /pix)	τ (ns)	*F0* (counts/pix)
TR	0.25	0.14	31.7	4.23	31.3
	0.50	0.28	57.5	4.07	61.2
	0.75	0.42	79.4	4.07	84.6
	1.00	0.57	93.1	3.93	106.3
	1.25	0.71	105.0	3.82	126.9
	1.50	0.85	112.0	3.59	153.3
NBD	0.25	0.21	22.1	6.99	22.2
	0.50	0.41	41.6	6.97	42.0
	0.75	0.61	56.3	6.98	56.6
	1.00	0.82	68.4	6.93	69.8
	1.25	1.02	74.0	6.67	81.5
	1.50	1.22	94.3	6.82	99.3
BODIPY	0.25	0.18	50.0	5.23	49.4
	0.50	0.37	68.8	5.09	71.8
	0.75	0.55	96.8	4.99	105.1
	1.00	0.73	120.0	4.78	142.0
	1.25	0.91	143.0	4.69	175.8
	1.50	1.09	148.0	4.54	194.2

Table S3 (caption on next page)

Table S3: *Calculation of the "non-quenched" fluorescence intensity as a function of fluorophore concentration for TR, NBD and BODIPY. The fluorophore concentration in weight/weight was converted to mole/mole using the molecular weights of each lipid, DOPC, TR-DHPE, NBD-DHPE and BODIPY-DHPE (786, 1382, 956 and 1067 g/mol, respectively). The raw fluorescence intensity, F, and the fluorescence lifetime,* τ*, were measured for each image (average of all pixels in one 25-frame FLIM acquisition). The non-quenched intensity,* F_0*, was calculated from F and* τ *using the equation:* $F_0 = F \cdot \exp\left[2\ln\left(\frac{\tau_0}{\tau}\right)\right]$.

SI 6. Generation of concentration profiles and quantifying quenching from FLIM images of lipid bilayers undergoing electrophoresis

To generate a concentration profile of the fluorophore distribution across a membrane corral, the conversion factors specified in the previous section (**SI 5**) were applied. Below, an example of the conversion process is described for TR (the data and related text is adapted from ref. (9)). This process was applied to all samples (TR, NBD, BODIPY, new data). **Figure S6A** shows a FLIM image of a membrane corral at electrophoretic equilibrium which had a starting concentration of 0.28% (mol/mol) TR, showing the direction of the profiles generated from left-to-right across the membrane (*white dashed region*). The mean values for fluorescence intensity and lifetime were calculated for each horizontal (x) position across the membrane corral by averaging vertical columns (y) of pixels. The resulting profiles for fluorescence lifetime and raw fluorescence intensity are shown in **Figure S6B-C** (*black* and *bright red* datapoints, respectively). The corrected fluorescence intensity (F_0) profile was calculated using main text Eq. 23, as described in the previous SI section (*dark red datapoints* in **Figure S6C**). The mole-to-mole TR fluorophore concentration at each horizontal position (*blue datapoints* in **Figure S6D**) was then calculated from F_0, as above. We found that the TR concentration in this example increased roughly exponentially from zero on the right side of the corral up to ~2.0% (mol/mol) at the left side of the corral. This was correlated to a decrease in the fluorescence lifetime of TR from ~4 ns to ~2.8 ns from right-to-left.

To compare the fluorescence quenching between TR, NBD and BODIPY this analysis was performed for all the membrane corral samples described in the main text. The profiles of raw fluorescence intensity and fluorescence lifetime are shown in **Figure S6E** and **Figure S6F**. As expected, all fluorophores show much higher fluorescence intensity at the left-edge of the corral correlated to a lowered fluorescence lifetime. The corrected fluorescence intensity and the calculation concentration profiles (**Figure S6G-H**). The maximum concentrations achieved at the left edge of the corral vary somewhat from 2-3% for TR and BODIPY to 4-6% for NBD due to different mole-to-mole starting concentrations for the different fluorophores. The important point is not the absolute concentration achieved but what degree of quenching is observed for that concentration. The amount of quenching can be quantified from the fluorescence lifetimes as the Quenching Efficiency ($QE = 1 - \tau/\tau_0$) and profiles were plotted of QE against distance (**Figure S6I**). A side-by-side comparison of the concentration profiles versus the QE profiles is instructive (**Figure S6**, panel **H** *vs.* **I**). It is clear that BODIPY becomes most quenched for its concentration compared to NBD which has much lower quenching despite reaching double the concentration; TR appears to have intermediate levels of quenching.

Whilst comparison of concentration profiles versus QE profiles is revealing, we decided that that it would be even more informative to directly compare the fluorophore concentration to the QE on the same graph. This would allow the concentration dependence of quenching to be assessed and related to the theoretical models, so that one could assess whether the transfer-to-trap mechanism is occurring. Therefore, scatter plots of QE against concentration were generated by using the fact that the line profiles described above are actually paired data, i.e., each location on a concentration profile has an observed value for fluorescence lifetime. Plots of QE against concentration are displayed in the main text as part of **Figure 4** and **Figure 5** and are explained and discussed there.

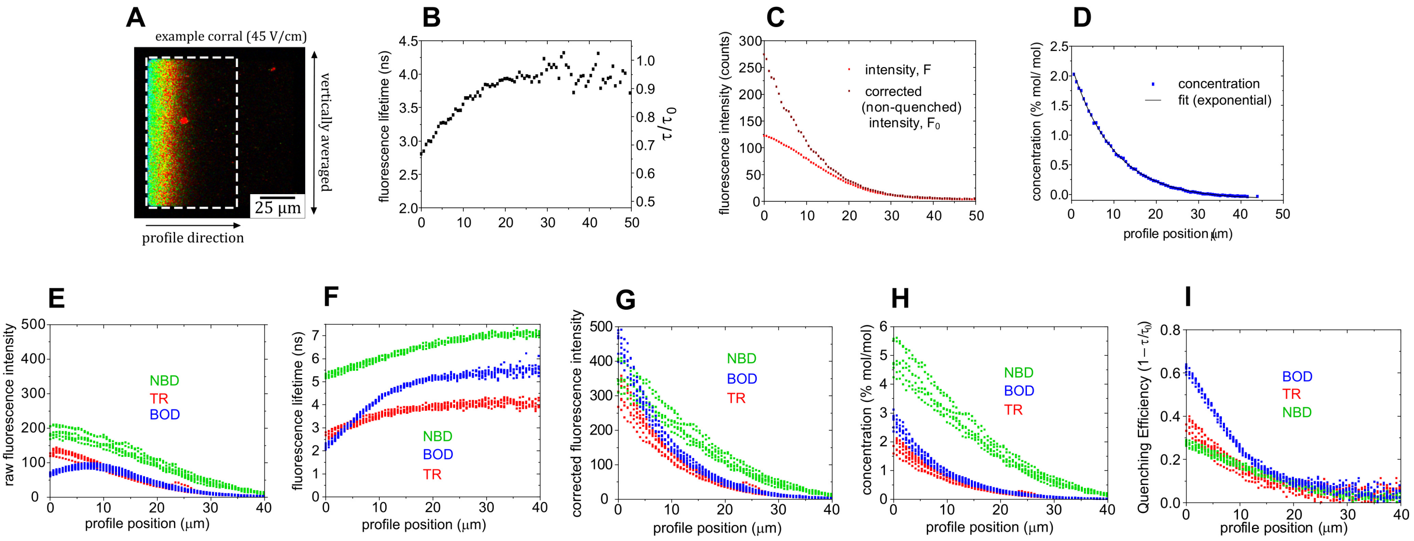

Figure S6: *Method for generating concentration profiles from a FLIM image applied to TR, NBD and BODIPY. **(A)** Example FLIM image of a 0.28% (mol/mol) TR corral at equilibrium in an E-field. The white dashed box denotes the region-of-interest (ROI) from which horizontal profiles of lifetime and intensity were obtained by averaging the pixels accumulated vertically (typically 150 pix/vertical, improving the signal-to-noise). **(B)** Profile of fluorescence lifetime against x-position obtained from the white dashed region in (A). **(C)** Profile of raw fluorescence intensity profile (light red), F, obtained from the white dashed region in (A). The corrected fluorescence intensity, F_0 (dark red), was calculated from the profile for* $F(x)$ *and the profile for* $\tau(x)$ *using Eq. 23 in the following form:* $F_0(x) = F(x) \cdot e^{2\,ln\,[\tau_0/\tau(x)]}$. ***(D)** The concentration profile (blue) calculated from the data for F_0 from (C) using the direct proportionality relationship between the molar concentration and non-quenched intensity of a fluorophore, C = F_0 / 168.3. The solid black line is a fit to mono-exponential function:* $C(x) = a \cdot e^{-bx} + y_0$ *(a, b and y_0 are fitting constants). **(E)** The raw fluorescence intensity profiles from a series of membrane corrals containing either TR, NBD or BODIPY fluorophores (colouring as labelled). Each profile is generated by vertically averaging the data, as shown in the example membrane corral in (A). Data from multiple independent membrane corrals is overlaid (n = 6 for each fluorophore type). **(F)** Fluorescence lifetime profiles corresponding to the fluorescence intensity profiles from (E). **(G)** Profiles of corrected fluorescence intensity as calculated from the profiles for* $F(x)$ *from (E) and the profiles for* $\tau(x)$ *from (F). Before calculating these profiles, a few counts of noise were subtracted from the raw fluorescence intensity by aligning all curves to F = 0 at x > 40 μm. **(H)** Concentration profiles as calculated from the data for F_0 from (G) using the direct proportionality relationships between the molar concentration and corrected intensity of a fluorophore, C = F_0 / 168.3, F_0 / 73.0, or F_0 / 168.8 for TR, NBD or BODIPY, respectively. **(I)** Profiles of Quenching Efficiency calculating from the equation:* $QE(x) = 1 - [\tau(x)/\tau_0]$ *using the corresponding fluorescence lifetime data from (F). Panels A-D are reproduced from Meredith et al. 2023 (9). All other data is new.*

SI 7. Comparison of the simplified versus exact mathematical models for quenching

To recap, the reduction in fluorescence intensity and lifetimes according to main text Eq. 6 and Eq.8:

$$\frac{\tau}{\tau_0} = 1 - P_{ETT} \qquad \text{main text Eq. 6}$$

and:

$$\frac{F}{F_0} = (1 - P_{ETT})(1 - f_T) \qquad \text{main text Eq. 8}$$

where f_T is the fraction of fluorophores involved in traps, P_{ETT} is the probability of excitation transfer to a trap site, F_0 is the original fluorescence intensity, F is the fluorescence intensity after quenching, τ_0 is the original fluorescence lifetime, τ is the fluorescence lifetime after quenching.

f_T depends only on the critical radius of trap formation (R_C) for a given fluorophore, at a given concentration (C_N). Whereas, P_{ETT} depends upon the FRET efficiency which, in turn, depends upon the Förster radius (R_0).

The exact model applies an expression for P_{ETT} based on the fluorophore concentration (C_N), the known value for Förster radius (R_0), and the critical radius of trap formation (R_C), so:

$$\frac{\tau}{\tau_0} = 1 - \left[\frac{R_0^6}{R_0^6 + (\pi C_N)^{-3}}(1 - e^{-\pi R_C^2 C_N})\right] \qquad \text{Eq. S36}$$

and:

$$\frac{F}{F_0} = \left\{1 - \left[\frac{R_0^6}{R_0^6 + (\pi C_N)^{-3}}(1 - e^{-\pi R_C^2 C_N})\right]\right\}\left\{e^{-\pi R_C^2 C_N}\right\} \qquad \text{Eq. S37}$$

Whereas, **the simplified model** assumes that $P_{FRET} = 1$, therefore $P_{ETT} = f_T$, and so:

$$\frac{\tau}{\tau_0} = e^{-\pi R_C^2 C_N} \qquad \text{main text Eq. 17}$$

and:

$$\frac{F}{F_0} = e^{-2\pi R_C^2 C_N} \qquad \text{main text Eq. 21}$$

At the end of this study, values for the critical radius for trap formation (R_C) were calculated from the experimental data of FLIM analysis of quenching during fluorophore electrophoresis (see main text **Figure 5**). These R_C values can be used to simulate the reduction in fluorescence intensity and the reduction in fluorescence lifetime that would occur according to the transfer to trap and statistical pair model, by solving both the exact models (Eq. S36 and Eq. S37) and the simplified model (Eq. 17 and Eq. 21) for a range of concentrations (C_N). The results are shown in **Figure S7**, below.

Here, we see the relative fluorescence intensity (F/F_0) decreases more rapidly with concentration than the relative fluorescence lifetime (τ/τ_0) and generally the trend lines for NBD > TR > BODIPY (**Figure S7A** vs **S7B**). There is a slight deviation between the trend produced by the exact model as compared to the simplified model at the lower concentrations, where the FRET assumption is invalid, and this deviation is most pronounced for NBD because it has the lowest Förster radius. Overall, these discrepancies are minor and equate to differences of 10% in the worst cases and the agreement between the two models is generally very good.

One subtle difference worth noting is that, according to the exact model, the fluorescence lifetime does not change appreciably below a fluorophore concentration of ~0.75% whereas the fluorescence intensity becomes somewhat quenched immediately. Now that we have estimates for R_0 and R_C for all three fluorophores, these graphs (solid lines in **Figure S7**) may be considered the most accurate representation of the expectations for fluorescence from these fluorophores.

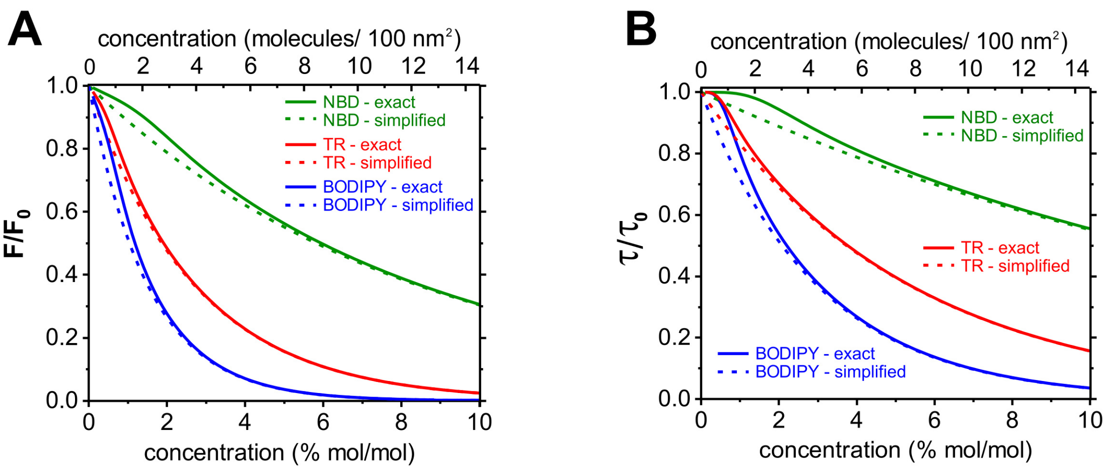

Figure S7: *Comparison of the exact and simplified theoretical models for transfer-to-trap quenching.* ***(A)*** *Relative fluorescence intensity as calculated from either Eq. S37 (exact) or Eq. 21 (simplified). This is proportional to the fluorescence quantum yield.* ***(B)*** *Relative fluorescence lifetime as calculated from either Eq. S36 (exact) or Eq. 17 (simplified). "Relative fluorescence lifetime" is sometimes termed the "fluorescence lifetime yield".*

SUPPLEMENTAL REFERENCE LIST

1. Beddard, G. S., S. E. Carlin, and G. Porter. 1976. Concentration quenching of chlorophyll fluorescence in bilayer lipid vesicles and liposomes. *Chem. Phys. Lett.* 43:27-32.
2. Kelly, A. R., and G. Porter. 1970. Model systems for photosynthesis I. Energy transfer and light harvesting mechanisms. *Proc. R. Soc. London, Ser. A* 315:149-161.
3. Chandrasekhar, S. 1943. Stochastic problems in physics and astronomy. *Rev. Mod. Phys.* 15:1-89.
4. Krider, E. P. 2013. The nearest neighbor probability distribution (University of Arizona, ATMO489, Atmospheric Electricity). http://www.atmo.arizona.edu/students/courselinks/spring13/atmo589/ATMO489_online/lecture_19/lect19_nearest_neighbor_dist.html (accessed 23 May 2024).
5. Jordan, D., and P. Smith. 2008. Mathematical techniques: An introduction for the engineering, physical, and mathematical sciences. Oxford University Press, Oxford, UK.
6. Martin, B. R., and G. Shaw. 2015. Mathematics for physicists. Wiley, Chichester, UK.
7. van Weerd, J., S. O. Krabbenborg, J. Eijkel, M. Karperien, J. Huskens, and P. Jonkheijm. 2014. On-chip electrophoresis in supported lipid bilayer membranes achieved using low potentials. *J. Am. Chem. Soc.* 136:100-103.
8. Bao, P., M. R. Cheetham, J. S. Roth, A. C. Blakeston, R. J. Bushby, and S. D. Evans. 2012. On-chip alternating current electrophoresis in supported lipid bilayer membranes. *Anal. Chem.* 84:10702-10707.
9. Meredith, S. A., Y. Kusunoki, S. D. Connell, K. Morigaki, S. D. Evans, and P. G. Adams. 2023. Self-quenching behavior of a fluorescent probe incorporated within lipid membranes explored using electrophoresis and fluorescence lifetime imaging microscopy. *J. Phys. Chem. B* 127:1715-1727.
10. Johansson, L. B. Å., and G. Lindblom. 1986. Application of time-resolved fluorescence in the study of lipid aggregates II. Motions and order of pyrene probes in an aligned lyotropic nematic phase. *Liquid Crystals* 1:53-62.
11. Wistus, E., E. Mukhtar, and L. B. Å. Johansson. 1996. A conspicuous orientation and localization of pyrene in Langmuir lipid monolayers. *Langmuir* 12:3371-3373.
